# Substrate specificity of human MCPIP1 endoribonuclease

**DOI:** 10.1038/s41598-018-25765-2

**Published:** 2018-05-09

**Authors:** Mateusz Wilamowski, Andrzej Gorecki, Marta Dziedzicka-Wasylewska, Jolanta Jura

**Affiliations:** 1 Department of General Biochemistry, Faculty of Biochemistry, Biophysics and Biotechnology, Jagiellonian University, Krakow, Poland; 2 Department of Physical Biochemistry, Faculty of Biochemistry, Biophysics and Biotechnology, Jagiellonian University, Krakow, Poland

## Abstract

MCPIP1, also known as Regnase-1, is a ribonuclease crucial for regulation of stability of transcripts related to inflammatory processes. Here, we report that MCPIP1 acts as an endonuclease by degrading several stem-loop RNA structures and single-stranded RNAs. Our studies revealed cleavage sites present in the stem-loops derived from the 3′ untranslated region of the interleukin-6 transcript. Furthermore, MCPIP1 induced endonuclease cleavage at the loop motif of stem-loop structures. Additionally, we observed that MCPIP1 could cleave single-stranded RNA fragments. However, RNA substrates shorter than 6 nucleotides were not further affected by MCPIP1 nucleolytic activity. In this study, we also determined the dissociation constants of full-length MCPIP1_D141N_ and its ribonuclease domain PIN D141N with twelve oligonucleotides substrates. The equilibrium binding constants (Kd) for MCPIP1_D141N_ and the RNA targets were approximately 10 nM. Interestingly, we observed that the presence of a zinc finger in the PIN domain increases the affinity of this protein fragment to 25-nucleotide-long stem-loop RNA but not to shorter ones. Furthermore, size exclusion chromatography of the MCPIP1 and PIN proteins suggested that MCPIP1 undergoes homooligomerization during interaction with RNA substrates. Our results provide insight into the mechanism of MCPIP1 substrate recognition and its affinity towards various oligonucleotides.

## Introduction

Ribonuclease degradation of mRNA is an essential mechanism to control the level of selected transcripts in cells. MCPIP1 (Monocyte Chemoattractant Protein-1–Induced Protein 1), also known as Regnase1, regulates RNA stability through its ribonucleolytic activity. Regulation of immune responses by MCPIP1 occurs through the direct degradation of transcripts of many cytokines, such as IL-1β, IL-2, IL-6, IL-8, IL-12b, and IL-17^1–7^. MCPIP1 was described as a modulator of inflammatory processes in the early phase of inflammation^5^. MCPIP1 also regulates differentiation, tumor growth and angiogenesis^8–10^.

The enzymatic activity of MCPIP1 is due to the PIN domain (PilT N–terminus), which possesses ribonucleolytic activity^1,2^. The putative MCPIP1 active site consist of four aspartate residues that are engaged in coordination of a single magnesium ion localized in the enzyme catalytic cleft^11^. PIN domains are commonly present in various eukaryotic and prokaryotic nucleases that cleave different classes of RNA molecules, including mRNA, rRNA, tRNA and viral RNAs^12,13^. One of those nucleases is the Dis3 subunit of the eukaryotic exosome complex, which contains a PIN domain that has endonuclease activity against mRNA. Additionally, the C-terminal domain of Dis3 possesses processive 3′ to 5′ exonuclease activity^14^. PIN domains are frequently present as the toxin agent of prokaryotic proteins engaged in toxin-anti-toxin systems, including the VapBC system containing the VapC PIN ribonuclease^15^. Recently, the *Caenorhabditis elegans* protein REGE-1 was shown to contain a functional nuclease PIN domain, indicating close homology to MCPIP1^16^.

MCPIP1, which is encoded by *ZC3H12A*, belongs to the MCPIP family comprising products of the genes *ZC3H12A, ZC3H12B*, *ZC3H12C* and *ZC3H12D*^17^. A specific feature shared by this family is a single CCCH zinc finger (ZF) domain positioned at the C-terminal region of the PIN ribonuclease domain. CCCH-type ZFs are characteristic of proteins involved in RNA processing. Several representatives of CCCH ZF RNA-binding proteins are tristetraproline (TTP), Roquin1 and Roquin2^18^. The CCCH ZF increases the efficiency of RNA substrate cleavage catalyzed by MCPIP1^11,19^. Additionally, the crystal structure of the PIN domain revealed the positively charged loop sequence that is located near the catalytic core of MCPIP1. This loop may mediate the interaction with negatively charged phosphate groups of oligonucleotide backbones^11^. Homooligomerization of MCPIP1 occurs through the C-terminal domain, which is enriched in proline residues. Deletion of this region decreased ribonucleolytic activity of MCPIP1^20^. Purified recombinant MCPIP1 protein with a mutation in the nuclease catalytic site (D141N) retained the ability to recognize RNA, and formation of the nucleoprotein complex was observed in gel shift electromobility assays^19,21^.

The half-life of transcripts is primarily modulated through RNA-binding proteins that recognize *cis*-regulatory elements, such as AU-rich elements (AREs) or stem-loop structures. MCPIP1 recognizes stem-loops in mRNA and degrades transcripts in an ARE-independent manner^1,2,4^. Analyses of sequences obtained from high-throughput sequencing of RNA isolated by crosslinking immunoprecipitation (HITS-CLIP) showed that stem-loop sequences preferably recognized by MCPIP1_D141N_ contain pyrimidine-purine-pyrimidine (YRY) loop motifs^5^. These results indicated that the MCPIP1 ribonuclease recognizes sequences present in certain structural motifs. Interestingly, MCPIP1 and Roquin cooperate in posttranscriptional gene regulation by processing the same set of target mRNAs^5,22^. The YRY sequence motif was also previously identified in targets recognized by Roquin1, which binds stem-loop RNA^23^. However, Roquin1 itself does not possess nuclease activity, and regulation of transcripts occurs through the recruitment of the CCR4-NOT deadenylase complex^24^.

Many transcripts that were determined in high-throughput sequencing analysis as a transcript negatively regulated by MCPIP1 do not possess the YRY motif in the loop structure of stem-loops. Moreover, some of these transcripts were also validated as targets for MCPIP1-induced degradation. It was shown that fragments derived from 3′UTR of the transcripts coding for interleukin-2 121–140, BCL2L1 and BIRC3 deprived of YRY motif in the stem loops are not targets for MCPIP1 induced degradation.4,9 Interestingly, MCPIP1 has potential to recognize the stem-loop sequences with a wide range of sizes. For example, the reported consensus sequence from HITS-CLIP analysis is 7 nt-3 nt-7 nt (stem-loop-stem)5. However, validated as a target for MCPIP1 the stem loop from 3′UTR transcript coding for mouse STAT3 1739–1765 contains 10 nt-8 nt-10 nt (stem-loop-stem) motif ^25^. Therefore, it is possible that MCPIP1 recognizes loop sequences with various nucleotide (nt) content and structures. Thus, in this study, we focused on describing the specificity of MCPIP1 substrate recognition using RNA cleavage assays and affinity determination assays.

## Results

### Determination of substrate specificity for MCPIP1

We purified recombinant human MCPIP1_WT_ and MCPIP1_D141N_, which were expressed in *E. coli* cells. The purity of the analyzed proteins was confirmed by SDS-PAGE analysis (Supplementary Fig. S1). To define the MCPIP1 nuclease substrate specificity, we performed RNase cleavage assays using 4 types of oligonucleotides: 17-25-nt-long RNA forming stem-loop structures (mIL-6_82–106_, mIL-6_85–101_ short stem, hIL-6_82–99_), 7-12-nt-long single-stranded RNA (mIL-6_82–93_, mIL-6_82–88_), 12-nt-long single-stranded DNA (mIL-6_82–93_ ssDNA) and 12-nt-long double-stranded DNA (mIL-6_82–93_ dsDNA). These sequences were derived from the 3′ untranslated region (UTR) of the IL-6 transcript. Furthermore, we also analyzed consensus stem-loop sequences that were previously identified as MCPIP1 targets^5^ and single-stranded poly-U RNA sequences. Detailed information about the applied oligonucleotide sequences is presented in Table 1.

**Table 1:** Nt sequences of fluorescently modified oligonucleotides used for the RNase assays and affinity determination assays.

Name	Sequence	Length
**RNA oligonucleotides**		
mIL-6_82–106_ 3′FAM	5′-UGUUGUUCUCUACGAAGAACUGACA-3′-FAM	25 nt
mIL-6_82–106_ 5′FAM	FAM-5′-UGUUGUUCUCUACGAAGAACUGACA-3′	25 nt
mIL-6_82–106_ RS	FAM-5′-**ACAGUCAAGA**CUACGA**UCUUGUUGU**−3′	25 nt
mIL-6_82–106_ YR	FAM-5′-UGUUGU**ACA**CUACGA**UGU**ACUGACA-3′	25 nt
mIL-6_85–101_ short stem	FAM-5′-UGUUCUCUACGAAGAAC-3′	17 nt
hIL-6_82–99_	FAM-5′-UGUUCUCUAUGGAGAACU-3′	18 nt
consensus stem-loop	FAM-5′-UGGAAAGUAUCUUUCCU-3′	17 nt
mIL-6_82–93_	FAM-5′-UGUUGUUCUCUA-3'	12 nt
mIL-6_82–88_	FAM-5′-UGUUGUU-3'	7 nt
poly-U	FAM-5′-UUUUUUUUUUUU-3'	12 nt
mIL-6_82–106_ int. ACA	FAM-5′-UGU**ACA**UCUCUACGAAGA**UG**U**U**ACA-3′	25 nt
mIL-6_83–98_ ter. ACA	FAM-5′-**ACA**GUUCUCUAUGGAGAAC**UGU**−3′	22 nt
mIL-6_85–93_ ter. ACA	FAM-5′-**ACA**UGUUCUCUA-3′	12 nt
mIL-6_1–45_	FAM-5′-UGCGUUAUGCCUAAGCAUAUCAGUUU GUGGACAUUCCUCACUGUG-3′	45 nt
**DNA oligonucleotides**		
mIL-6_82–93_ ssDNA	FAM-5′-TGTTGTTCTCTA-3′	12 nt
mIL-6_82–93_ dsDNA	FAM-5′-TGTTGTTCTCTA-3′ 3′-ACAACAAGAGAT-5′	12 bp

Nts that form loop fragments of stem-loop structures are underlined. Sequences with numbered residues are part of the 3′UTR of transcripts from mouse or human IL-6. These sequences were numbered such that the first nt after the stop codon of the coding sequence is marked as 0. RS – reverse stem modification of mIL-6_82–106_ (altered nts are in bold). YR – purine and pyrimidine residue modification of mIL-6_82–106_ (altered nts are in bold)_._

Because the activity of MCPIP1 is dependent on the presence of Mg^2+^ or Mn^2+^ metal ions, all degradation assays were performed in buffer with the divalent cation Mg^2+^. Additionally, to decrease non-specific electrostatic interactions between MCPIP1 and nucleic acids, the cleavage studies were performed at physiological salt concentration (150 mM NaCl). The observed MCPIP1 ribonuclease activity products were reproducibly consistent for proteins obtained from different batches.

**Figure 1:**
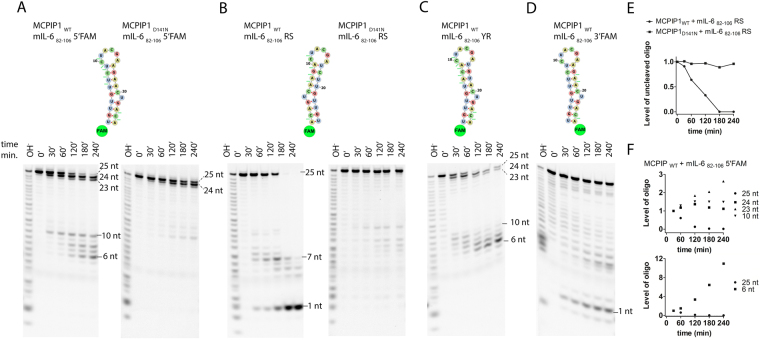
RNA fragments obtained upon MCPIP1-catalyzed cleavage. (**A**) Sequences and structures of the 25-nt-long RNA stem-loops derived from the mIL-6_82–106_ 3′UTR fragment are depicted. (**B)** Degradation assay of the modified mIL-6_82–106_ stem-loop (RS – reverse stem alteration). (**C**) Degradation assay of the modified mIL-6_82–106_ stem-loop (YR – pyrimidine and purine alterations). (**D)** Degradation assay of the mIL-6_82–106_ stem-loop labeled at the 3′ end. The oligonucleotides were labeled at the 5′ or 3′ end with FAM dye. The reaction products were resolved on 20% denaturing PAGE and visualized with a fluorescence imaging system. Concentrations of the labeled oligonucleotides and MCPIP1 protein were 7.5 μM and 2 μM, respectively. The D141N mutation of a conserved aspartate of the PIN domain catalytic center of the MCPIP1 decreased its ribonucleolytic activity. (**A**–**D**) Were made by the separation of gels that are shown at Supplementary Fig. S2A,D. Major sites of MCPIP1-induced cleavage are indicated by the oligonucleotide fragment length. (**E**) Densitometric analysis of the kinetics of RNA degradation presented as the level of the remaining uncleaved oligonucleotides from the RNase assay. The graph shows the level of the stem-loop sequence: mIL-6_82–106_ RS during degradation. (**F**) Densitometric analysis of the MCPIP1_WT_ induced degradation of the mIL-6_82–106_ 5′FAM oligonucleotide. Analysis was carried out separately for each of degradation products of the mIL-6_82–106_ 5′FAM. Oligonucleotide levels were normalized at the time 30 min.

In each case, the RNA cleavage assay was carried out for 30, 60, 120, 180 and 240 minutes. We initially performed an RNase assay of the mIL-6_82–106_ stem-loop structure. We observed that MCPIP1_WT_ induced degradation starting from the 3′ end of the mIL-6_82–106_ 5′FAM, and MCPIP1_WT_ cleaved the 25^th^ single nt as the first one. Then, the 24^th^ nt was cleaved (Fig. 1A). Simultaneously, mIL-6_82–106_ 5′FAM stem-loop cleavage occurred at the loop site, between the C10 and U11 nts (Fig. 1A). In the consequence, a 10 nt single-stranded RNA fragment was generated from the 5′ end of the mIL-6_82–106_ 5′FAM stem-loop structure. Next, additional processive degradation of the nascent 10-nt-long ssRNA was observed (Fig. 1A). However, MCPIP1-induced degradation was not observed for ssRNA fragments consisting of 6 nt (Fig. 1A).

Next, to verify the stereospecificity of MCPIP1-induced cleavage, we reversed the sequence of the mIL-6_82–106_ at the stem site of this stem-loop. Surprisingly, after reversing the stem sequences (mIL-6_82–106_ RS oligonucleotide), we observed a single nt product induced by MCPIP1_WT_ activity, indicating that enzymatic hydrolysis occurred between first (A) nt and the second (C) nt (Fig. 1B). Thus, the reverse stem sequence (mIL-6_82–106_ RS) was cleaved between the same nts as the basic mIL-6_82–106_ stem-loop, in which MCPIP_WT_ triggered enzymatic hydrolysis between A_25_ and C_24_ nt in the vicinity of the 3′ end of the oligonucleotide (Fig. 1A,B). Moreover, accumulation of the 7-nt-long degradation product of the mIL-6_82–106_ RS oligonucleotide indicated that MCPIP1_WT_ introduced cleavage between the A_7_ and A_8_ nts (Fig. 1B).

We also examined whether the analysis of oligonucleotide degradation was affected by potential *E. coli* contaminants remaining from the protein purification procedure. Therefore, we analyzed oligonucleotide cleavage induced by MCPIP1_D141N_ with a substitution of the conserved aspartate at the catalytic center of the PIN domain. No ribonucleases activity of the MCPIP1_D141N_ was observed for the mIL-6_82–106_ RS stem-loop oligonucleotide (Fig. 1B,E). However, low-efficacy nuclease activity of MCPIP1_D141N_ was observed for mIL-6_82–106_ 5′FAM, as shown in Fig. 1A. MCPIP1_D141N_ induced cleavage occurred only at 3′ end of this oligonucleotide. Thus, the D141N mutation does not completely abolish *in vitro* enzymatic activity of MCPIP1.

The characteristic feature of the unmodified mIL-6_82–106_ stem-loop is a high presence of pyrimidine residues at the 5′ site of the stem. Therefore, to assess the role of this characteristic pattern, we modified the stem sequence to achieve balanced distribution of the purine and pyrimidine residues at the stem site of this stem-loop. Nts 6–9 and 16–19 were changed in mIL-6_82–106_ YR (Table 1 and Fig. 1C). Our results showed that MCPIP1_WT_-induced degradation of the mIL-6_82–106_ YR occurs at the same time at the loop site of the stem-loop structure or at the 3′ end of the stem-loop structure (Fig. 1C). Furthermore, we determined that after destabilization of the mIL-6_82–106_ YR stem loop structure through loop cleavage induced by MCPIP1_WT_, the 10-nt-long ssRNA was increased and subsequently processively degraded (Fig. 1C). Additionally, we observed that degradation of mIL-6_82–106_ YR stops at the fragment consisting 6 nt, similar to the degradation of the unmodified mIL-6_82–106_ 5′FAM oligonucleotide (Fig. 1A,C). Alteration of purine with pyrimidines (mIL-6_82–106_ YR) did not change the cleavage sites in the stem loop structure, and degradation was triggered as in the case of mIL-6_82–106_ 5′FAM (Fig. 1A,C). Thus, we concluded that MCPIP1_WT_-induced *in vitro* degradation is not dependent on stem sequence of the stem-loop.

To avoid negative results due to diminished cleavage susceptibility of sites where nts are modified by fluorescent labeling, we labeled the mIL-6_82–106_ stem-loop structure at the 5′ end or at 3′ end (mIL-6_82–106_ 5′FAM, mIL-6_82–106_ 3′FAM, respectively). For the mIL-6_82–106_ 3′FAM sequence, we observed a 1-nt-long degradation product; therefore, the first cleavage induced by MCPIP1_WT_ occurs between C_24_ and A_25_ nts as shown in Fig. 1D. Thus, cleavage between C_24_ and A_25_ was observed for both the mIL-6_82–106_ 5′FAM and mIL-6_82–106_ 3′FAM sequences. However, for the 3′FAM-labeled oligonucleotide, the degradation was less efficient. Comparison of Fig. 1A,D suggests that the presence of fluorescent dye on a cleaved nt does not significantly affect the MCPIP1 activity.

### Kinetics of oligonucleotide degradation triggered by MCPIP1 depends on many factors

**Figure 2:**
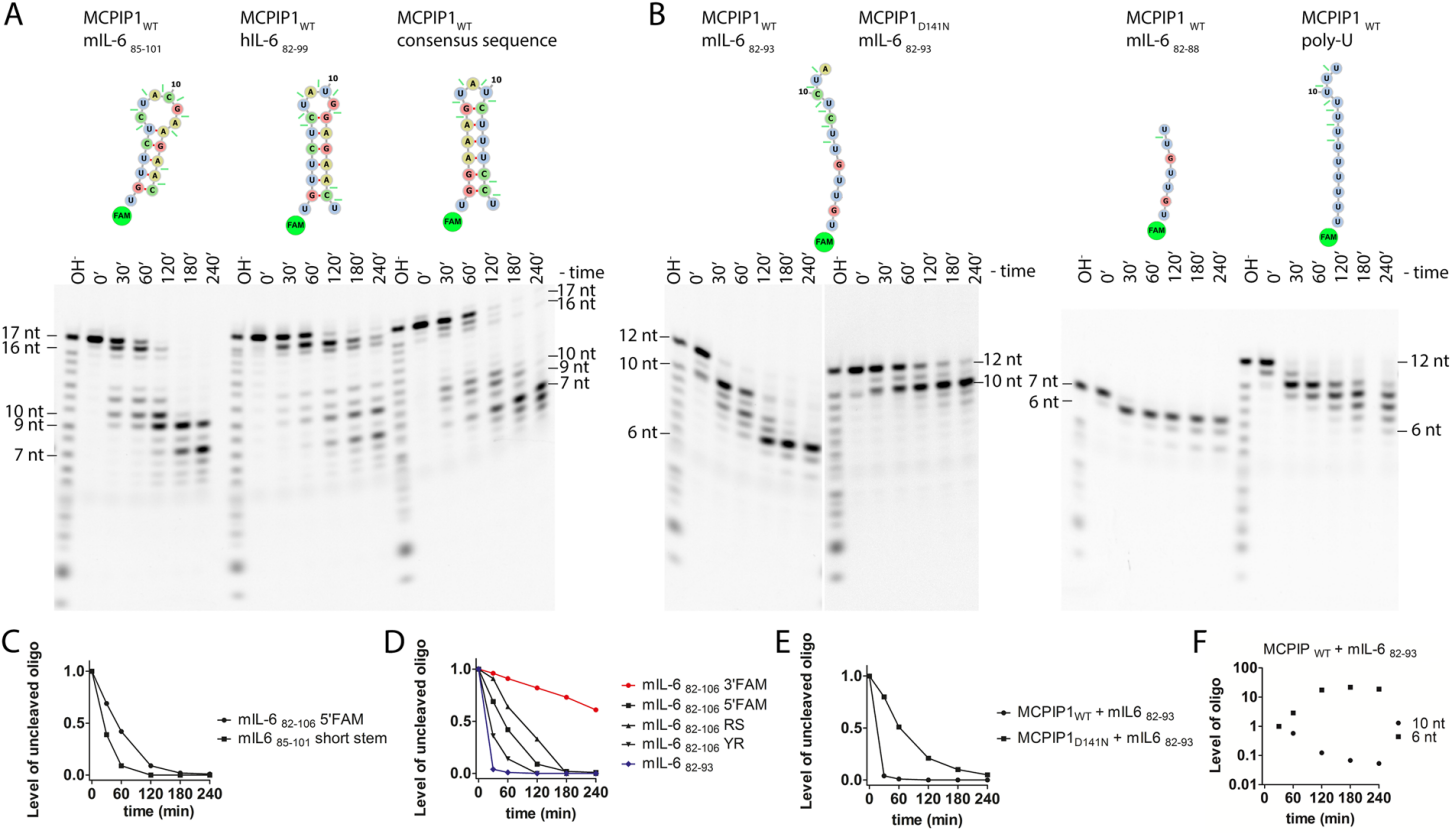
RNA fragments obtained upon cleavage catalyzed by MCPIP1. Major sites of MCPIP1-induced cleavage are indicated by the oligonucleotide fragment length. (**A**) Sequences and structures of the short stem-loops are depicted. These sequences form 17-18-nt-long stem-loops. (**B**) Degradation of the single-stranded RNAs. Twelve and 7-nt-long single-stranded RNA sequences were part of the mIL-6_82–106_ stem-loop. Poly-U homopolymer RNA contains 12 uracil residues. The oligonucleotides were labeled at the 5′ end with FAM dye. The reaction products were resolved on 20% denaturing PAGE and visualized with a fluorescence imaging system. Concentrations of the labeled oligonucleotides and MCPIP1 protein were 7.5 μM and 2 μM, respectively. The D141N mutation of a conserved aspartate of the PIN domain catalytic center of the MCPIP1 decreased its ribonucleolytic activity. (**A**,**B)** Were made by the separation of gels that are shown at Supplementary Fig. S2B–D. (**C**) Densitometric analysis of the level of the remaining uncleaved oligonucleotides from the RNase assay at different time points. The panel shows a comparison of the kinetics of degradation of the mIL-6_82–106_ and a shorter fragment of this stem-loop, which is mIL-6_85–101_. (**D**) The panel shows the level of degradation of the 25-nt-long stem-loop mIL-6_82–106_ in comparison with the 12 nt ssRNA sequence of the mIL-6_82–93_. (**E**) The graph compares the degradation of the ssRNA mIL6_82–93_ with MCPIP1_WT_ or MCPIP1_D141N_, which possesses attenuated RNase activity. (**F**) Densitometric analysis of the MCPIP1_WT_ induced degradation of the mIL-6_82–93_ oligonucleotide. The analysis was carried out for 6 nt and 10 nt long mIL-6_82–93_ degradation products respectively, oligonucleotide levels were normalized at the time 30 min.

In the next step, we examined whether the stem-loop structure, oligonucleotide length or nucleotide sequence affected MCPIP1 nucleolytic efficiency. To verify the impact of different stem-loop structures and sequences on MCPIP1-triggered cleavage, we used a set of short stem-loops consisting of 17 or 18 nts (mIL-6_85–101_, hIL-6_82–99_, consensus stem-loop) (Table 1). We observed that MCPIP1_WT_ induced cleavage of two nts from the 3′ end of these oligonucleotides and also accumulation of bands that are 10, 9 and 7 nt long (Fig. 2A). These findings indicated that MCPIP1_WT_ introduces endonucleolytic cleavage in the loop region of those short stem-loops (mIL-6_85–101_, hIL-6_82–99_, consensus stem-loop). Initial MCPIP1_WT_-induced enzymatic hydrolysis occurs simultaneously for four phosphodiester bonds between 9–12 nt at the loop motif of the mIL-6_85–101_ sequence (Fig. 2A). We determined that the pattern of loop cleavage of the mIL-6_85–101_ stem-loop is different than that for mIL-6_82–106_ 5′FAM, which was cut between the C10 and U11 nts (Fig. 1A). Therefore, the stem length of the stem-loop affects the cleavage sites recognized by MCPIP1.

To verify the influence of size of high-order RNA backbone structures on the oligonucleotide cleavage rates, we performed kinetic analysis. The kinetics of oligonucleotide degradation are shown as the level of uncleaved oligonucleotides obtained from densitometric analysis of the results from oligonucleotide degradation assays. In the subsequent time points of the RNase assay, uncleaved mIL-6_85–101_ oligonucleotide levels were significantly decreased compared to uncleaved levels of the mIL-6_82–106_ oligonucleotides (Fig. 2C). We observed that MCPIP1_WT_-triggered cleavage of the mIL-6_85–101_ stem-loop was relatively faster than that of the hIL-6_82–99_ stem-loop, which possesses longer stems (Fig. 2A). These results showed that the kinetics of degradation of RNA stem-loop structures containing short stems is faster than that of stem-loops possessing longer stems. We concluded that unwinding of shorter stems from the stem-loop structures results in a more efficient degradation (Figs 1A and 2A,C). To determine the importance of loop fragments in MCPIP1-triggered stem-loop cleavage, we compared stem-loops that contain a 3, 4 or 6 nt long loop motif. However, we did not observe major differences in MCPIP1-induced degradation of these oligonucleotides (Fig. 2A).

Subsequently, we assessed whether MCPIP1_WT_ degrades unstructured ssRNA. After MCPIP1_WT_-triggered destabilization of stem-loop structures, a subsequent cleavage occurred in the nascent ssRNA. Thus, we examined 12-nt-long ssRNA oligonucleotides from the mIL-6_82–93_ and 7-nt-long mIL-6_82–88_ ssRNA (Fig. 2B). Using the RNA folding software mFOLD^26^, we confirmed that the mIL-6_82–93_ and mIL-6_82–88_ sequences did not show base pairing interactions at room temperature; thus, they do not fold into stable secondary structures. We noticed that for the unstructured ssRNA, the rate of MCPIP1_WT_-induced degradation was increased compared to cleavage of the stem-loop sequences (Fig. 2D). Degradation of either mIL-6_82–93_ or mIL-6_82–88_ indicated that ssRNAs shorter than 6 ribonucleotides were not efficiently cleaved by MCPIP1_WT_ (Fig. 2B). The levels of shortened oligonucleotides formed as a result of the MCPIP1_WT_ induced cleavage of the mIL-6_82–106_ 5′FAM indicated high increase of the 6 nt long truncated oligonucleotide (Fig. 1F). Furthermore, we observed 11-fold increase of the level of 6 nt product of the MCPIP1_WT_ induced cleavage of the mIL-6_82–93_ (Fig. 2F). Moreover, there was marginal catalytic activity of MCPIP1_D141N_ for ssRNA, which presented as cleavage of two nts from the 3′ end of the mIL-6_82–93_ oligonucleotide (Fig. 2B,E). To verify the sequence specificity of ssRNA cleavage, we performed degradation assays using poly-U sequences. However, it appeared that MCPIP1_WT_ processively cleaved the poly-U homopolymer, and the oligonucleotide degradation stopped when the fragment consisted of 6 nt (Fig. 2B). These results indicated that MCPIP1_WT_ cleaves unstructured ssRNA in a sequence-independent manner.

**Figure 3:**
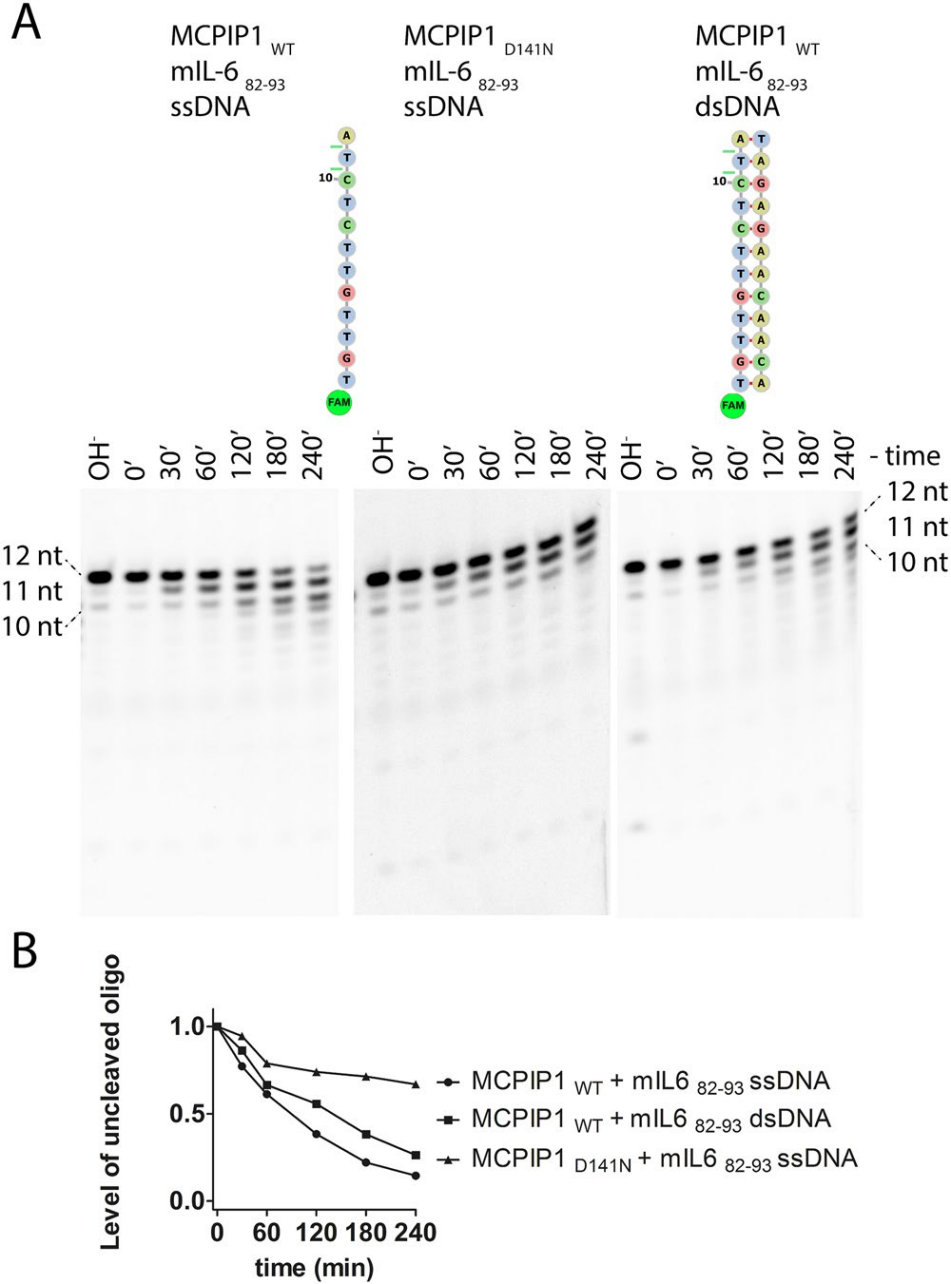
DNA fragments obtained upon cleavage catalyzed by MCPIP1. (**A**) The DNA sequences are based on the mIL-6_82–106_ sequence; one oligonucleotide is ssDNA, and the second is dsDNA. The oligonucleotides were labeled at the 5′ end with FAM dye. The reaction products were resolved on 20% denaturing PAGE and visualized with a fluorescence imaging system. Concentrations of the labeled oligonucleotides and MCPIP1 protein were 7.5 μM and 2 μM, respectively. The D141N mutation of a conserved aspartate of the PIN domain catalytic center of the MCPIP1 decreased its ribonucleolytic activity. (**A**) Was made by the separation of gels that are shown at Supplementary Fig. S2C,D. (**B**) Densitometric analysis of the level of the remaining uncleaved ssDNA and dsDNA oligonucleotides from the MCPIP1 nuclease activity assay at different time points.

We next investigated whether MCPIP1 exhibits RNA substrate specificity. Therefore, in RNase cleavage assays, we used single-stranded and double-stranded DNA (ssDNA and dsDNA) as a substrate. These DNA sequences were similar to RNA sequences consisting of 12 nts present in the mIL-6_82–93_ oligonucleotide. We observed that MCPIP1_WT_ cleaves both ssDNA and dsDNA (Fig. 3A). Degradation of these sequences occurred from the 3′ end; however, the kinetics of these processes was lower compared with the cleavage of mIL-6_82–93_ ssRNA (Figs 2B,E and 3A,B). Degradation of the mIL-6_82–93_ ssDNA had approximately equal efficiency using either MCPIP1_WT_ or MCPIP1_D141N_ (Fig. 3A,B). Therefore, the aspartate 141 residue of MCPIP1 is crucial for RNA cleavage but not for DNA processing (Figs 1A,B, 2B and 3A,B).

We showed that MCPIP1_D141N_ does not possess activity against mIL-6_82–106_ RS (Fig. 1B). However, we have observed that MCPIP1_D141N_ possesses low nuclease activity in some of the investigated systems (Figs 1A, 2B and 3A). Therefore, to further confirmation that presented RNA cleavage assay is not affected by contaminations from *E. coli* extract we used another control which is MCPIP1_438–599_ protein deprived of PIN nuclease domain. Applying MCPIP1_438–599_ to RNase assay we did not observe degradation of investigated oligonucleotides (Supplementary Fig. S2E). Thus, our results are not affected by contaminations and we conclude that single mutation D141N of MCPIP1 is not sufficient to completely abolish *in vitro* MCPIP1 nuclease activity. All identified cleavage sites observed in degradation assays are listed in Supplementary Table S1. To verify the sequence specificity of MCPIP1-triggered degradation of RNA, we presented the identified sites of cleavage as a consensus logo (Supplementary Fig. S3). For logotype preparation, we used the sequence logo generator software WebLogo^27^. We figured out that cleavage sites lacking G nts in the immediate vicinity of the cut site were preferable for MCPIP1-induced cleavage (Supplementary Fig. S3).

Observed at Fig. 1 oligonucleotides cleavage patterns and results presented at Supplementary Fig. S3 revealed possible MCPIP1 sequence specificity within 5′-ACA-3′ motif. To confirm this observation we prepared three additional oligonucleotides that contain 5′-ACA-3′ modifications (Table 1). The internal modification of stem sequence to 5′-ACA-3′ was introduced to the mIL-6_82–106_ (Supplementary Fig. S2F). We observed that MCPIP1_WT_ induced cleavage of the mIL-6_82–106_ int. ACA occurs at loop site between the C10 and U11 nts, then, additional cleavages were spotted between A4-C5-A6 nts (Supplementary Fig. S2F). However, incorporation of terminal 5′-ACA-3′ to the hIL-6_83–98_ sequence revealed that for this oligonucleotide MCPIP1_WT_ induced cleavage takes place between sequences U5-U6-C7 (Supplementary Fig. S2F). For single stranded RNA addition of terminal 5′-ACA-3′ to the mIL6_85–93_ showed that MCPIP1_WT_ induces hydrolysis of bond between C2 and A3 nts of the mIL6_85–93_ ter. ACA (Supplementary Fig. S2F). Nevertheless, processive 3′ to 5′ cleavage of single stranded RNA is highly efficient compared to endonuclease cleavage (Supplementary Fig. S2F).

To further confirmation of our observation about *in vitro* nonspecific cleavage of RNA oligonucleotides by MCPIP1_WT_ we performed additional experiments. We checked whether MCPIP1_WT_ might cleave the template which were previously reported at *in vivo* studies as not degraded by MCPIP1 nuclease activity. The fragment comprising 1–81 nt from the mIL-6 3′UTR is not regulated through MCPIP1 activity in cells studies^5^. The distal part of the mIL-6 3′UTR contains the mIL-6_82–106_ 3′UTR stem loop which is the putative element responsible for IL-6 transcripts destabilization through MCPIP1 nuclease activity. Due to limitations of synthesis methodology we used 45 nts long sequence from the mIL-6_1–45_ 3′UTR (Table 1 and Supplementary Fig. S2F). Using RNA folding software mFOLD^26^ we showed that the mIL-6_1–45_ oligonucleotide possibly forms two stem-loops structures as shown at Supplementary Fig. S2F. Degradation of the mIL-6_1–45_ 3′UTR clearly indicates endonuclease activity of MCPIP1_WT_. The fragment of the mIL-6_1–45_ tends to form two stem-loop secondary structures, thus observed cleavage induced by MCPIP1_WT_ should be introduced at loop site of these stem loops. Indeed we observed endonuclease cleavage of the mIL-6_1–45_ at both loop sites (Supplementary Fig. S2F). However, due to obtained low electrophoresis resolution we could not precisely describe the exact nucleotides between which cleavage takes place (Supplementary Fig. S2F).

### Dissociation constants of the MCPIP1 complex with oligonucleotides

Our results from oligonucleotide degradation assays did not reveal a strong structural or sequence preference of *in vitro* RNA cleavage by recombinant MCPIP1_WT_. However, we determined that single-stranded RNA or 17-nt-long stem-loops were cleaved with a faster rate than 25-nt-long stem loops. For that reason, we investigated whether there were any differences in MCPIP1_D141N_ affinity for the tested oligonucleotides. We used FAM-labeled oligonucleotides to develop a method for determination of the MCPIP1_D141N_ affinity to oligonucleotides. Previously, we used electrophoretic mobility shift assays (EMSAs) to show that MCPIP1_D141N_ has the potential to form stable complexes with 3′UTR fragments of the C/EBPβ transcript and obtained complexes possessing two distinct quaternary structures^21^. Observed shifts at EMSA assay indicated that the marginal nuclease activity of MCPIP1_D141N_ did not repress formation of the nucleoprotein complex. Estimated binding affinities of the complexes of MCPIP1_D141N_ with RNA based on our results published previously by Lipert *et al*. were between 640–1580 nM (Supplementary Table S2)^21^. The obtained Kd varies from previously used the 3′UTR sequence fragments of the C/EBPβ transcript. However, in our previous EMSA experiments, we were not able to determine the equilibrium dissociation constants of the achieved complexes.

**Figure 4:**
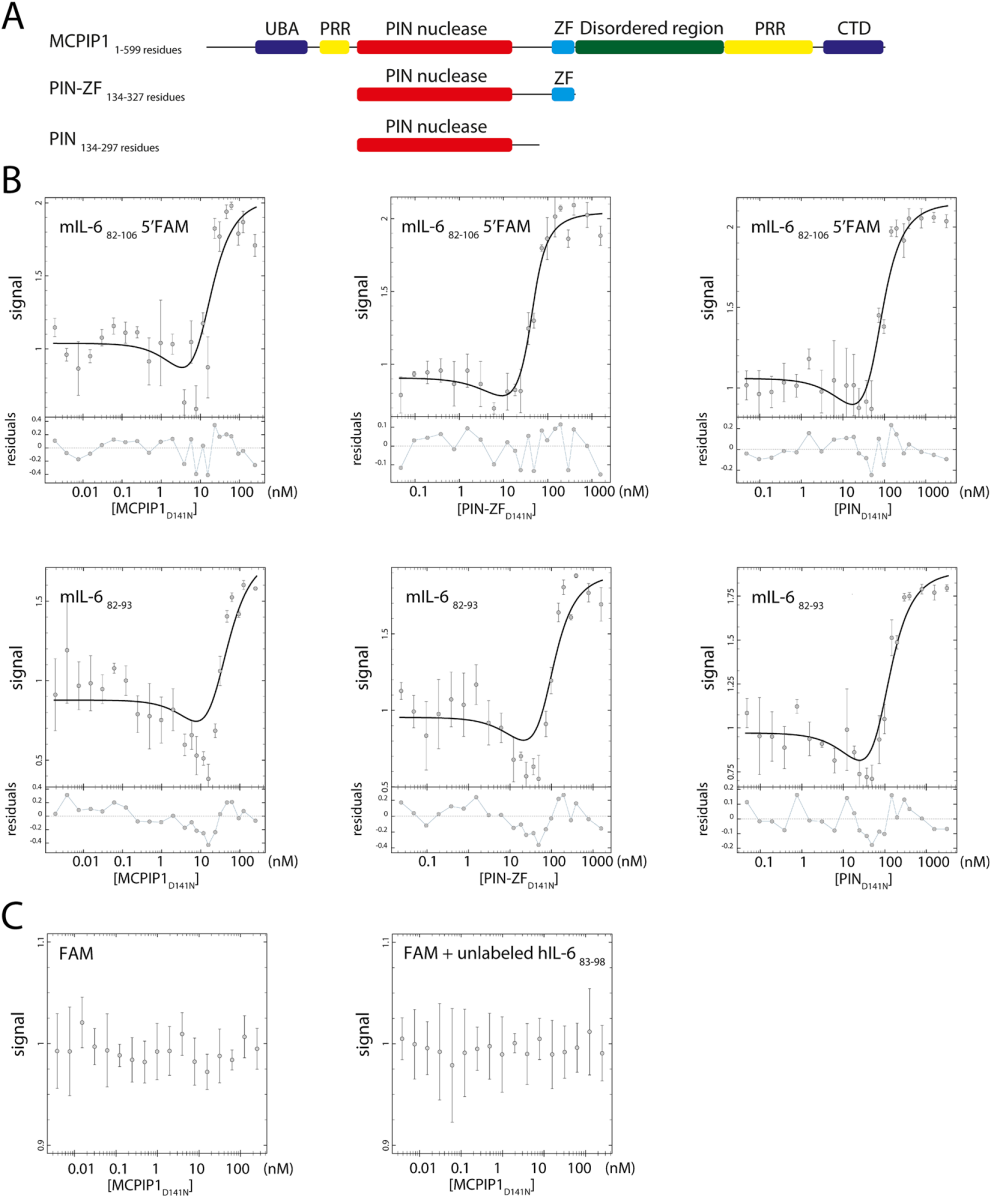
(**A**) Domain characterization of MCPIP1: UBA_43–89_ (Ubiquitin-associated domain); PRR_100–126_ and _458–536_ (Proline-rich region); PIN_133–270_ (PilT N-terminus nuclease domain); ZF_305–325_ (zinc-finger motif); disordered region_326–457_; CTD_545–598_ (C-terminal conserved domain). Depicted fragments of MCPIP1, PIN-ZF and PIN that were used in presented studies. (**B**) Affinity of the MCPIP1 interaction with oligonucleotides forming RNA stem-loop structures: mIL-6_82–106_ 5′FAM and single-stranded RNA oligonucleotides represented by mIL-6_82–93_. The analyzed proteins were the catalytic mutated forms: MCPIP1_D141N_ and its PIN-ZF_D141N_ and PIN_D141N_ fragments. The ribonuclease PIN_D141N_ domain was studied without or with the zinc finger motif at the C-terminal region. Graphs illustrate the interaction of selected proteins (MCPIP1_D141N_, PIN-ZF_D141N_, and PIN_D141N_) with oligonucleotides. Functions were fitted to the fluorescence intensity data points using the sequential binding model N + P + P $\rightleftarrows$ NP + P $\rightleftarrows$ NPP (P – protein N – oligonucleotide). The depicted errors bars are standard deviations, n = 3. (**C**) Controls of the affinity determination assay. MCPIP1_D141N_ in a presence of the free FAM label and unlabeled hIL-6_81–98_ RNA oligonucleotide.

Herein, we determined the apparent equilibrium dissociation constants of the human MCPIP1_D141N_ complexes with different types of oligonucleotides: stem-loop RNA, ssRNA, ssDNA and dsDNA (Fig. 4B, Supplementary Fig. S4 and Table 2). The slopes of dose-response curves were very steep for protein concentration values between 10 nM and 100 nM. Amplitudes of the fluorescence signals were changed approximately 2 times depending on the sequence. Fluorescence polarization assay which is commonly used for affinity determination might be affected by high fluorescence intensity changes observed in our measurements. Thus, we decided to analyze only fluorescence intensity signal which also gives us better residuals of obtained fits. The two-phase course of fluorescence intensity changes, observed in all investigated cases, prompted us to model the affinity data with a complex double equilibrium binding equation where N + P + P $\rightleftarrows$ NP + P $\rightleftarrows$ NPP (N – oligonucleotide, P – protein) (Fig. 4B and Supplementary Fig. S4). These results showed that two protein molecules sequentially bind to a single oligonucleotide. The best model describing our data was a sequential binding model with equal equilibrium-binding dissociation constants, K_d1_ = K_d2_. We also analyzed the model characterized by K_d1_≠K_d2_, which was rejected because it inconsiderably improved the residual distribution, however, the standard deviations of the calculated Kd were higher than those for the model with Kd_1_ = Kd_2_. A third analyzed model with a single equilibrium constant, N + P + P $\rightleftarrows$ NPP, was rejected because it had the highest residuals of curves that were fitted to the measured data points. The final selected model (sequential binding analysis, K_d1_ = K_d2_) reflects the measured data well, and the obtained K_d_ are shown in Table 2. This model was characterized by the lowest standard deviation of the obtained dissociation constant values and low residuals of the fitted curves.

**Table 2:** Calculated equilibrium dissociation constants of MCPIP1 complexes with selected oligonucleotides.

	MCPIP1_D141N_ 1–599 aa K_d_ (nM)	PIN-ZF_D141N_ 134–327 aa K_d_ (nM)	PIN_D141N_ 134–297 aa K_d_ (nM)
**Stem-loop RNAs**			
mIL-6_82–106_ 5′FAM	6.5 ± 2.1^a^	6.6 ± 3.1^b^	24.1 ± 7.3^ab^
mIL-6_82–106_ 3′FAM	8.6 ± 2.6^a^	4.8 ± 1.8^b^	21.8 ± 6.3^ab^
mIL-6_82–106_ RS	9.8 ± 2.3^a^	15.1 ± 7.3^b^	39.3 ± 8.6^ab^
mIL-6_82–106_ YR	9.5 ± 5.2^a^	14.4 ± 9.6	25.1 ± 7.0^a^
mIL-6_85–101_ short stem	9.2 ± 4.0^a^	20 ± 10	20.7 ± 7.2^a^
hIL-6_82–99_	4.1 ± 3.6^a^	13.2 ± 6.8	15.1 ± 4.5^a^
consensus stem-loop	3.8 ± 2.8^a^	9.1 ± 3.2^b^	36 ± 17^ab^
**ssRNA**			
mIL-6_82–93_	19 ± 11^a^	34 ± 18	41 ± 12^a^
mIL-6_82–88_	14.5 ± 8.1^a^	32 ± 20	40 ± 12^a^
poly-U	14.8 ± 7.2^a^	43 ± 23	39 ± 11^a^
mIL-6_82–93_ ssDNA	18.3 ± 8.2	36 ± 20	21.7 ± 6.6
mIL-6_82–93_ dsDNA	118 ± 49	80 ± 30	56 ± 17

The analyzed proteins were mutated forms of MCPIP1 and its ribonuclease domain (MCPIP1_D141N_, PIN-ZF_D141N_, and PIN_D141N_). K_d_ values were determined using DynaFit4 software with the implemented model of sequential binding of two proteins to a single oligonucleotide with a single dissociation constant. Errors are shown as standard deviations, n = 3. Statistical significance (P value < 0.05) between selected groups is shown by the following indexes: ^a^ and ^b^ for comparison of the MCPIP1_D141N_ with PIN_D141N_ and PIN-ZF_D141N_ with PIN_D141N_ groups, respectively. Differences observed between the MCPIP1_D141N_ and PIN-ZF_D141N_ groups are not statistically significant.

We observed that for the set of investigated oligonucleotides comprising stem-loop structures, ssRNA, and ssDNA, we did not find major differences between dissociation constants of the complexes with MCPIP1_D141N_ (Table 2). Therefore, MCPIP1_D141N_ can efficiently bind diverse oligonucleotide sequences. Minor differences in the MCPIP1_D141N_ affinity to stem-loop structures or single-stranded oligonucleotides suggest that the nucleic acid double-stranded helical structure is not necessary to interact with MCPIP1. Additionally, we observed that MCPIP1 has lower affinity to dsDNA comparing to other investigated nts (Table 2). We showed that the affinity of full-length MCPIP1_D141N_ to oligonucleotides is significantly higher than that for fragments of this protein represented only by the nuclease domain (PIN_D141N_) (Fig. 4A,B, Supplementary Fig. S4 and Table 2). Moreover, we noticed that the zinc finger domain increased the affinity of the PIN_D141N_ subunit to 25-nt-long oligonucleotides but not to shorter oligonucleotides (Fig. 4A,B, Supplementary Fig. S4 and Table 2).

Binding assays using free FAM dye did not showed significant changes of fluorescence intensity at investigated systems (Fig. 4C). MCPIP1_D141N_ and buffer condition did not affect fluorescence emission of the free FAM label. The unlabeled hIL-6_81–98_ RNA oligonucleotide did not affect fluorescence emission of free FAM label in the presence of MCPIP1_D141N_ (Fig. 4C).Thus, we assume that described interactions are the effect of the assembly of the MCPIP_D141N_ complex with oligonucleotides. The shape of fluorescence spectra of the FAM labeled oligonucleotides were consistent for all examined MCPIP_D141N_ concentrations (Supplementary Fig. S4A). Fluorescence intensity of the FAM labeled oligonucleotides were changed due to MCPIP1_D141N_ nucleoprotein complex formation which affected FAM fluorescence probe (Supplementary Fig. S4A).

Observed dissociation constants for MCPIP1_D141N_ complexes with mIL-6_82–106_ 5′FAM were substantially weaker for EMSA system than in fluorescence based assay (Table 2, Supplementary Fig. S5 and Supplementary Table S2). We suppose that differences in dissociations constants are the results of the complex binding kinetics of MCPIP1 interaction with RNA that possibly is characterized by relatively fast k_off_ rates. In case of high k_off_ the EMSA as a non-equilibrium method will give higher dissociation constants compared to equilibrium techniques. The EMSA shift for mIL-6_82–93_ ssRNA and mIL-6_82–93_ ssDNA were observable at a relatively low concentration of MCPIP_D141N_ (400 nM) (Supplementary Fig. S5A) although, at higher concentration of the MCPIP1_D141N_ the oligonucleotides were not completely bounded in nucleoprotein complex. Therefore, we didn’t calculate the Kd from that results. EMSA results might suggest that there are differences in quaternary structures of the complex between MCPIP1 D141N and different types of substrates (Supplementary Fig. S5A).

### Homooligomerization of MCPIP1

**Figure 5:**
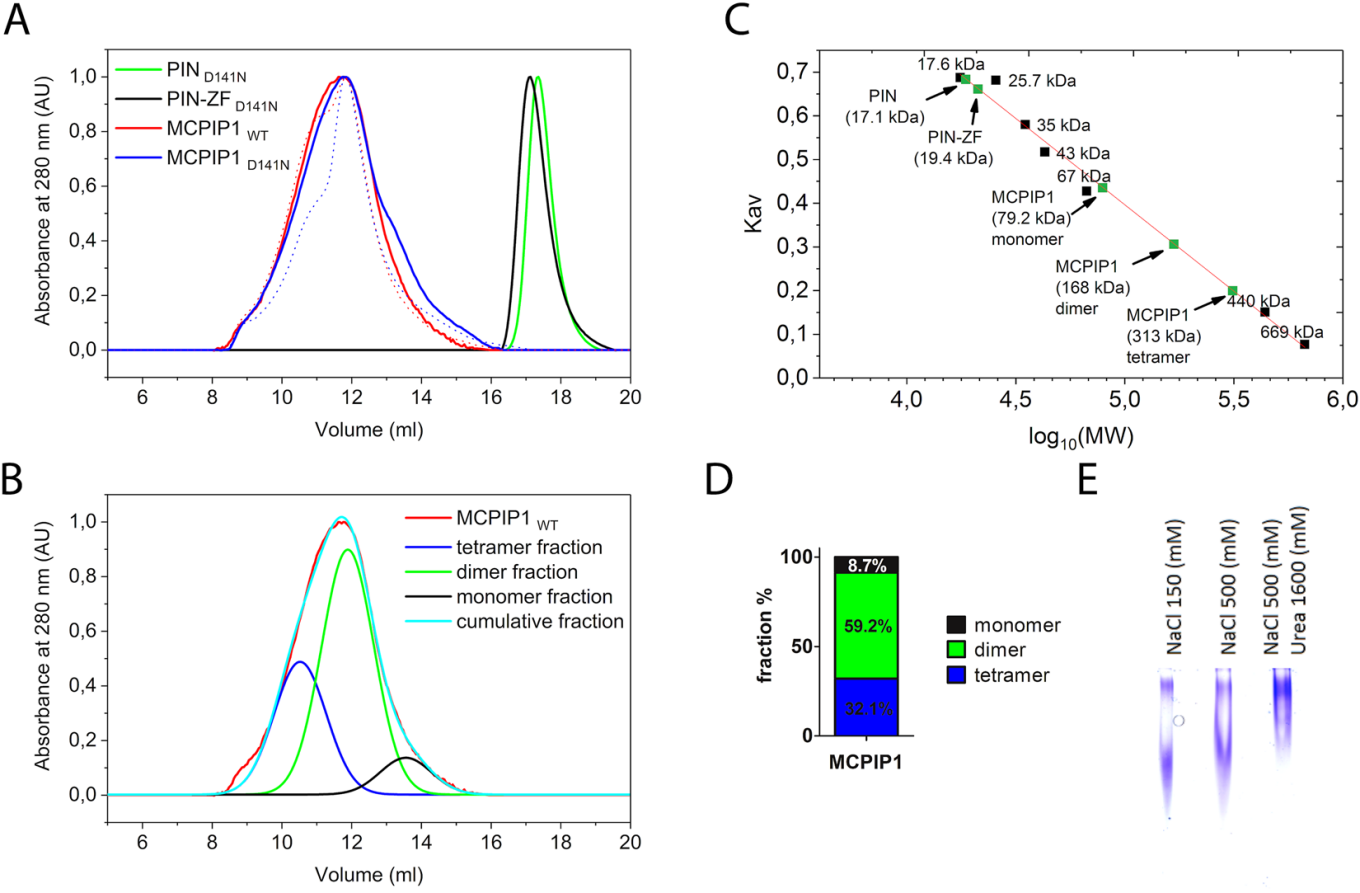
Homooligomerization of the MCPIP1 protein. (**A**) Size exclusion chromatography results of MCPIP1. Chromatography was performed in a buffer comprised of 25 mM Tris, pH 7.9, 300 mM NaCl, 10% (w/v) glycerol, 1 mM DTT, and 0.5 mM EDTA. Additionally, for results shown as a dotted line, the buffer was enriched in 1.6 M urea. (**B**) A multiple Gaussian peak fit was performed to model the obtained elution profile of MCPIP1_WT_. Fitted peaks illustrated tetrameric, dimeric and monomeric fractions of the MCPIP1_WT_. (**C**) Calibration curve of the gel filtration column. Green points indicate the apparent molecular weight of the investigated proteins calculated using the calibration curve. The molecular weights of these proteins are as follows: MCPIP1: 65.7 kDa; PIN-ZF: 24.7 kDa; PIN: 21.1 kDa. (**D**) Percentages of the MCPIP1_WT_ tetrameric, dimeric, and monomeric fractions were calculated based on the area of the size exclusion chromatography peaks. (**E**) Native PAGE results of MCPIP1_WT_ sample in buffers containing 50 mM Tris-HCl, pH 8.3, 150 mM NaCl, 10% (w/v) glycerol, 2.5 mM MgCl_2_, 1 mM DTT, 0.5 mM EDTA and 0.05 mM ZnCl_2_. Additional buffer condition changes were an increased concentration of NaCl to 500 mM and addition of urea to 1600 mM.

Interestingly, the two-phase course of fluorescence intensity changes was observed in the obtained affinity assay graphs during oligonucleotide-binding processes (Fig. 4B, Supplementary Fig. S4). Thus, two protein molecules sequentially bind to a single RNA molecule. According to other studies, PIN domain superfamily proteins are frequently described as oligomers: dimers or tetramers^28^. Therefore, to obtain precise data of the MCPIP1 protein oligomerization state, we analyzed protein size exclusion chromatography results. In both cases, single Gaussian peaks were observed, indicating the monodispersity of the analyzed protein fragments. Analysis of protein size based on the retention volume (Fig. 5A,C) indicates that both the PIN and PIN-ZF domains were in a monomeric state. The mouse PIN domain was previously suggested to be a dimer^19^. In contrast, for full-length MCPIP1_WT_ and MCPIP1_D141N_, we observed wide elution peaks that shifted in favor of possible oligomeric forms (Fig. 5A). To assess these elution profiles, we performed multiple Gaussian peak fit analyses (Fig. 5B). Comparing the obtained maxima of fitted peaks with the column calibration curve, we observed that the calculated molecular masses of the fractions corresponded to tetrameric, dimeric and monomeric forms of MCPIP1_WT_ (Fig. 5C,D). Therefore, the full-length MCPIP1_WT_ or MCPIP1_D141N_ is most frequently present as a dimer in native conditions, however, tetrameric and monomeric fractions were also present in solution (Fig. 5A–D). Buffer supplementation with 1.6 M urea resulted in narrowing peaks in size exclusion chromatography, indicating the additional increase of the MCPIP1 dimers fraction in the sample (Fig. 5A–D). Native PAGE electrophoresis of MCPIP1 revealed two heterogeneous bands for MCPIP1_WT_ (Fig. 5E). These results confirmed that MCPIP1 homooligomerization occurs in native conditions. Equilibrium between the dimeric and tetrameric states of MCPIP1 could be influenced by changing the buffer composition. After addition of urea to the native PAGE buffer, the equilibrium was shifted in favor of the MCPIP1 tetrameric fraction (Fig. 5D). Urea as a chaotropic agent alternates hydrogen bonding between water molecules and proteins, and therefore affect hydrophobic interactions^29^. The small concentration of urea or other commonly used denaturation agent: guanidine hydrochloride increases stability of some proteins and might increase reactions rates^30^. Urea concentration (1.6 M) used in our experiment has a much lower concentration than typically is used for denaturation of proteins, however, the concentration of this osmolyte is sufficient to significantly change the content of bulk water^29^. Urea can substantially influence the polypeptides solvation, increasing protein solvent accessible area which might consequently lead to conformation changes of MCPIP1 and probably its might affect oligomerization^30^. Although, this indirect mechanism appears to be the most likely in our case, there are reports indicating a possible alternative mechanism, in which the urea molecules directly interacts with protein molecules in a divalent manner^31^.

The two distinct quaternary structure of MCPIP1 D141N nucleoprotein complexes were also observed in EMSA, using long UTRs fragments as well as single stem loop of the mIL-6_82–106_, as shown by Lipert at al. in Supplementary Fig. 5^21^. Together, the size exclusion chromatography and native PAGE results indicate that the both dimeric and tetrameric forms of MCPIP1 homooligomers were found in the investigated conditions.

## Discussion

**Figure 6:**
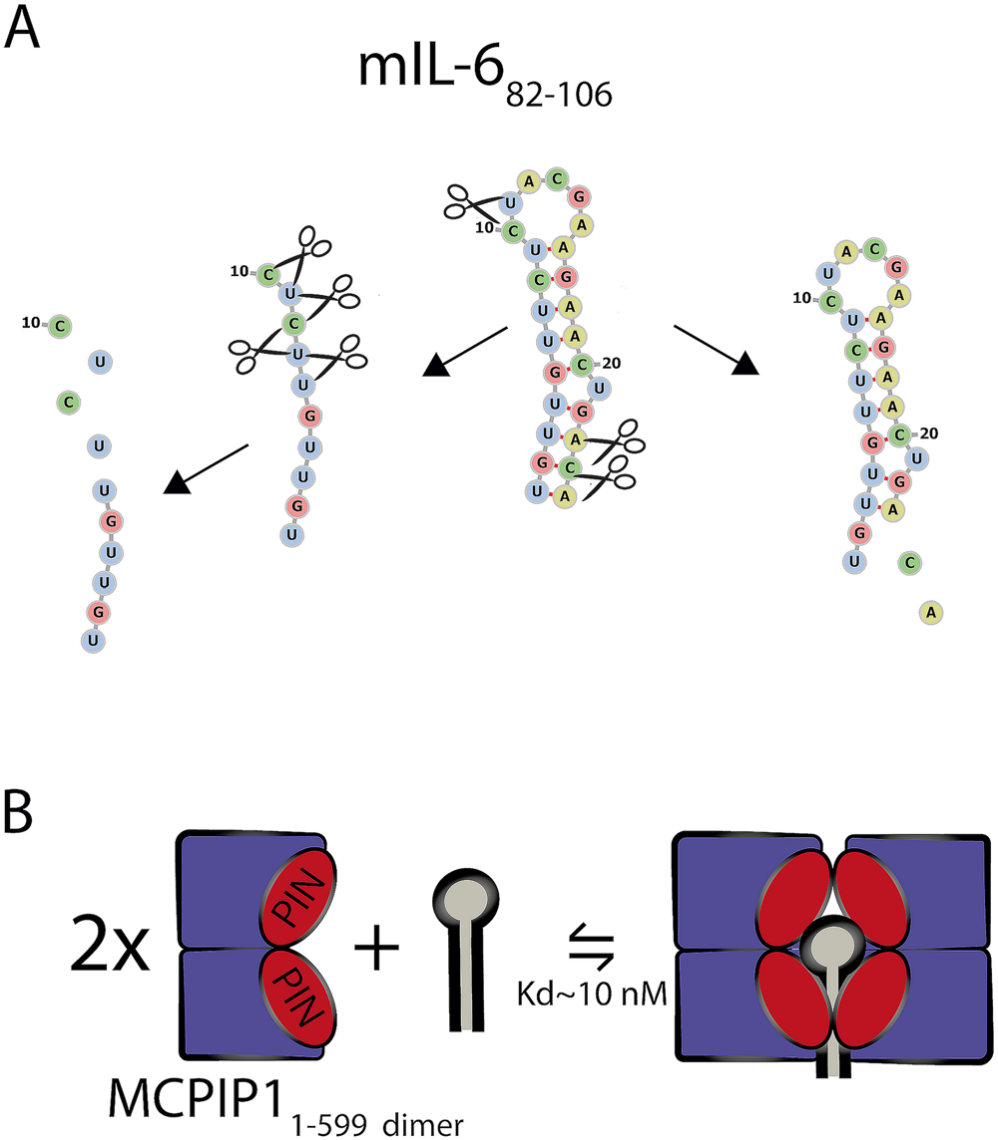
(**A**) Identification of MCPIP1-triggered cleavage sites in the mIL-6_82–106_ stem-loop RNA structure. Nt sequences and structures created during MCPIP1-induced cleavage are illustrated. Mapping of the cleavage sites based on the RNase assay results, intermediates and the most significant subsequent degradation products are presented. (**B**) Visualization of the stoichiometry of the MCPIP1 interaction with stem-loops. Schematic cartoon representation of the ternary complex model. The size exclusion chromatography results showed that PIN and PIN-ZF were monomeric and suggest that full-length MCPIP1 most frequently occurs as a dimer in native condition. Stoichiometry of the MCPIP1 - RNA interaction was based on the size exclusion chromatography results and the results from affinity determination assays where the sequential binding model were used. Thus, for full-length MCPIP1, we proposed a sequential binding model: oligo + MCPIP1_Dimer_ + MCPIP1_dimer_ $\rightleftarrows$ oligo-MCPIP1_dimer_ + MCPIP1_dimer_ $\rightleftarrows$ oligo-MCPIP1_tetramer_. The presented dissociations constants of the complexes were estimated based on the affinity determination assays shown in Table 2.

Previous studies indicated that MCPIP1 is a selective ribonuclease that cleaves translationally active mRNA at the 3′UTR^5^. To determine how MCPIP1 recognizes the unique molecular targets that were reported in biological systems, we applied *in vitro* analysis using recombinant MCPIP1. We identified MCPIP1 as an endoribonuclease that degrades diverse sets of RNA stem-loop structures. Collectively, our data did not indicate a strong structural or sequence preference during *in vitro* cleavage of the RNA stem-loops, as all investigated sequences were affected by MCPIP1. However, our results revealed that unstructured single-stranded RNA is highly prone to cleavage by MCPIP1_WT_. We observed that MCPIP1_WT_-induced degradation of the mIL-6_82–106_ stem-loop starts from the 3′ end of the sequence. Simultaneously, cleavage occurs at the loop site of the stem loop. As a consequence of loop cleavage, the stem-loop structure is destabilized, and ssRNA fragments are generated, which are further processively degraded in the next step (Fig. 6A). Surprisingly, we observed that 6-nt-long ssRNA was not rapidly cleaved by MCPIP1_WT_. A possible explanation for this process might be that the 6 nt RNA substrate is too short to reach the nuclease site. We hypothesized that the region of MCPIP1 that is crucial for RNA binding must be proximal to the catalytic cleft in the PIN domain since the 7-nt-long mIL-6_82–88_ substrate was still bound with high affinity to the PIN_D141N_ domain. We hypothesized that the positively charged region that is present in the structure of the MCPIP1 PIN domain is essential in the RNA recognition process and protects bound fragments of short ssRNA from further cleavage. Preferential cleavage of oligoribonucleotides triggered by MCPIP1 was observed for sequences lacking a G nt at positions −1 and +1 of the cleavage site, however, this observation might be affected by the low complexity of the analyzed sequences.

Degradation of 3′FAM-labeled oligonucleotides indicated that introduction of the fluorescent label did not disable the recognition of the cleavage sites by MCPIP1_WT_. However, for 3′FAM-labeled oligonucleotides, we observed a decrease in the nucleolytic efficiency of MCPIP1_WT_. Moreover, we observed that MCPIP1 cleaved poly-U ssRNA oligonucleotides in a processive manner. These findings may suggest 3′ to 5′ exonuclease activity of MCPIP1 against ssRNA. Additionally, previous reports revealed that the MCPIP1 PIN domain shares high structural homology with the T5 D15 5′-exonuclease^11,32^. Nevertheless, the successive exonuclease degradation of single-stranded RNA by MCPIP1 is not relevant *in vivo* due to the low rate of observed 3′ to 5′ exonuclease cleavage activity. Moreover, *in vitro* endonuclease activity of recombinant MCPIP1 had a strong background as shown in the results of cleavage of the loop sites of the investigated stem-loops. Additionally, previous results have also shown degradation of longer transcripts, such as IL-6, IL-8 or CEBPβ, which indicated that preferable sites of endonucleolytic cleavage are present in these transcripts^5,6,21^. The endonuclease activity of recombinant MCPIP1 was also confirmed from *in vitro* degradation of circular RNA fragments^5^.

We hypothesized that the marginal *in vitro* activity observed here of either MCPIP1_WT_ or MCPIP1_D141N_ towards DNA molecules is irrelevant *in vivo*, since MCPIP1 has a primary cytoplasmic localization and should be considered as a ribonuclease. In contrast, the EndoV nuclease efficiently cleaves both RNA and DNA substrates^33^. There are also evidences of nuclear localization of the MCPIP1 for which essential is nuclear localization signal (RKKP) that is present in amino acid sequence of the MCPIP1^34^. However, observed here DNA cleavage was inefficient thus we estimate DNAse activity of MCPIP1 as biologically insignificant.

Ribonucleases possessing PIN domains usually lack strong sequence specificity in *in vitro* studies with recombinant proteins^13^. However, protein engineering can modify the specificity of these RNases. One example is the engineered PIN-PUF nuclease that possesses a high sequence specificity of RNA degradation^35^. Most likely, modification of the PIN domain from MCPIP1 will enhance its specificity and will be beneficial for the development of a highly sequence-specific molecular tool.

To the best of our knowledge, the equilibrium dissociation constants of the complex of MCPIP1 with oligonucleotides have not been previously described. To investigate oligonucleotides, we determined the K_d_ values of the complex with MCPIP1_D141N_, PIN-ZF_D141N_ and PIN_D141N_. The dissociation constant studies revealed a high affinity of MCPIP1_D141N_ to oligonucleotides, however, they did not show a major difference in affinity parameters using different oligonucleotides. We observed that the affinity of MCPIP1_D141N_ and its fragments towards ssRNA, ssDNA and dsDNA substrates is lower than that for oligonucleotides forming stem loops. Moreover, we did not observe significant differences between the affinity of MCPIP1_D141N_ or PIN-ZF_D141N_ to the investigated oligonucleotides. Therefore, we hypothesized that the PIN-ZF fragment is crucial for maintaining the complex with oligonucleotides. We also observed that the zinc-finger domain significantly increased the affinity of the PIN_D141N_ domain to 25-nt-long oligonucleotides. Interestingly, for shorter oligonucleotides, we did not observe significant differences in the affinity for PIN_D141N_ or PIN-ZF_D141N_. We hypothesized that the zinc finger does not reach short substrates, which were localized in proximity to the catalytic pocket of MCPIP1. A zinc finger tethered in the vicinity of the PIN catalytic domain might enhance the re-association of the substrate and facilitate subsequent cleavage. In contrast, previous data suggested that long RNA fragments derived from the C/EBPβ 3′UTR mRNA interact with full-length MCPIP_D141N_ but not with PIN-ZF_D141N_^21^. We hypothesized that our previous results might be affected by the non-equilibrium conditions of the EMSA method. It is also possible that for maintaining of PIN-ZF_D141N_ interaction with long mRNA fragments, additional domains of MCPIP1 are crucial for the stability of the ternary complex.

Size exclusion chromatography results indicate that MCPIP1 exists in equilibrium between the dimeric and tetrameric state, we proposed a stoichiometric model of the MCPIP1 - RNA interaction. Two molecules of the MCPIP1 dimer interact with a single stem-loop structure (Fig. 6B). Thus, for the full-length MCPIP1, we proposed a sequential binding model: oligo + MCPIP1_dimer_ + MCPIP1_dimer_ $\rightleftarrows$ oligo-MCPIP1_dimer_ + MCPIP1_dimer_ $\rightleftarrows$ oligo-MCPIP1_tetramer_. We hypothesized that the binding of oligonucleotide substrates induces homooligomerization of the MCPIP1. Tetrameric oligomerization of the PIN domain was previously shown for Nob1p and PAE2754, where the PIN domains of these proteins form a ring structure with a central hole that is wide enough to accommodate ssRNA or ssDNA but not double-stranded oligonucleotides^36,37^. Nevertheless, further validation of this model of MCPIP1-RNA complex stoichiometry should be performed. Our model is based on size exclusion chromatography results and double binding equilibrium observed in the affinity curves. Resolving the quaternary structure of MCPIP1 with RNA oligonucleotides is crucial for understanding its detailed mechanism of RNA regulation. To date, there is no resolved holoenzyme structure of any of the PIN domains; thus, further studies of MCPIP1 complexes are highly interesting.

We observed increased rates of degradation of hairpins with short stems, which are consistent with results published by Mino and coworkers from HITS-CLIP analyses where small stem-loops that contained a 3-nt- or 4-nt-long loop motif were predominantly identified in complexes with MCPIP1_D141N_^5^. Recent studies have revealed the importance of UPF1 for cytoplasmic mRNA decay catalyzed by MCPIP1^5^. UPF1 is an RNA helicase that participates in degradation of mRNAs with premature termination codons that are crucial for nonsense-mediated mRNA decay (NMD)^38^. SMG6, one of the key proteins for NMD, also contains a PIN domain at the C-terminus that is responsible for ribonucleolytic activity and mRNA turnover^39^. For *in vitro* RNA substrate cleavage induced by recombinant SMG6, the presence of additional proteins with helicase activity is not necessary. We showed that MCPIP1 alone is sufficient to unwind and degrade substrates with stem-loop secondary structures *in vitro*. Nevertheless, UPF1 might enhance unwinding of MCPIP1 substrates as an RNA helicase since we observed that degradation of more stable (with low Gibbs free energy) stem-loops was less efficient. Interaction with UPF1 and other proteins may increase the rate of degradation of selected RNA targets and broaden the recognition potential of the MCPIP1 complex.

Our biochemical studies revealed numerous cleavage sites introduced by recombinant MCPIP1 in the investigated sequences. We found that MCPIP1 induced endonuclease cleavage in the loop motif of stem-loop structures. We hypothesized that the presence of strong and non-sequence-specific interactions with RNA would enable MCPIP1 to efficiently search for the stem-loop elements in transcripts, and identification of a stem-loop would result in endonucleolytic cleavage and transcript destabilization. Nevertheless, MCPIP1 has been identified as selective ribonuclease that cleaves translationally active mRNAs at the 3′UTR. This raises the possibility that additional proteins that are elements of a ternary complex consisting of transcripts and MCPIP1 might determine the final MCPIP1 specificity.

## Methods

### Cloning and protein purification

The human *ZC3H12A* gene that encodes MCPIP1 was optimized for efficient expression in *E. coli* strains and ordered as a synthetic gene from GenScript (USA). The cloning, expression and purification of the full-length MCPIP1_WT_ protein and its mutant form MCPIP1_D141N_ were previously described^6^. The procedures for purification of the N-terminus His_6_-tagged proteins PIN-ZF_WT_ and PIN-ZF_D141N_ (134–327 residues) were described previously^21^. The same methods were used to purify PIN_WT_ and PIN_D141N_ (134–297 residues), which were also tagged with His_6_ at the N-terminus. Briefly, *E. coli* BL21-CodonPlus-RIL cultures were grown at 37 °C in LB medium until reaching an OD_600_ of 0.5. Protein expression was induced with addition of 0.5 mM IPTG. All proteins were expressed for 3 hours at 37 °C. Full-length MCPIP proteins were purified using ion-exchange chromatography (TMAE) in denaturing conditions. PIN_WT_, PIN_D141N_, PIN-ZF_WT_ and PIN-ZF_D141N_ were purified using Ni-NTA affinity chromatography in denaturing conditions. Finally, all proteins were dialyzed and purified using a gel filtration Superdex 200 prep grade 10/300 (GE Healthcare) column in a buffer comprised of 25 mM Tris, pH 7.9, 300 mM NaCl, 10% (w/v) glycerol, 1 mM DTT, and 0.5 mM EDTA. Chromatography was performed using an Äkta FPLC purification system (Amersham Pharmacia).

### Fluorescent-labeled nucleic acid sequences

The oligonucleotide sequences listed in Table 1 were purchased from Sigma-Aldrich. These oligonucleotides were fluorescently labeled using 6-carboxyfluorescein (6-FAM). The 5′ ends labeling of oligonucleotides were made by attaching 6-FAM to phosphate group of the 5′ terminal nucleotides. The mIL-6_82–106_ 3′FAM labeling was done by coupling 6-FAM to phosphate group of the 3′ terminal nucleotide. Labeled and purified with high-performance liquid chromatography oligonucleotides were purchased from Sigma-Aldrich. The double-stranded mIL-6_82–93_ dsDNA was prepared by mixing mIL-6_82–93_ ssDNA and the complementary oligonucleotide in a 1:1.2 ratio. Subsequently, for dsDNA, oligonucleotides were annealed by heating to 95 °C for 5 minutes and cooled at room temperature. Analysis of RNA secondary structure of the investigated oligonucleotides was performed using the Vienna RNA web server^40^.

### RNase assays

*In vitro* cleavage assays of FAM-labeled oligonucleotides were performed in buffer containing 25 mM Tris-HCl, pH 7.9, 150 mM NaCl, 10% (w/v) glycerol, 2.5 mM MgCl_2_, 1 mM DTT, 0.5 mM EDTA and 0.05 mM ZnCl_2_. Labeled oligonucleotides and MCPIP1 protein concentrations were 7.5 µM and 2 µM, respectively. Samples were incubated at 37 °C, and reactions at different time points were stopped by freezing in dry ice. After addition of twofold excess of concentrated loading dye consisting of 95% (w/v) formamide, 0.5 mM EDTA, 0.025% (w/v) xylene cyanol, and 0.025% (w/v) bromophenol blue, reactions products were denatured at 95 °C for 1 minute. An alkaline hydrolysis RNA ladder for each oligonucleotide was generated through denaturation at 95 °C for 25 minutes in alkaline buffer containing 50 mM sodium bicarbonate, pH 9.5, and 1 mM EDTA. Samples were resolved in denaturing gel electrophoresis in TBE (Tris/borate/EDTA) buffer. Denaturing gels contained 20% polyacrylamide and 7.5 M urea. Fluorescence signals were detected using ChemiDoc gel imaging device with ImageLab 5.2 software (BioRad Laboratories). Signal acquisition times 0.5 sec were the same for each of the gels.

### Affinity determination assays

The concentration of FAM-labeled oligonucleotides was 2 nM in a system with MCPIP1_D141N_ and 20 nM in a system containing PIN_D141N_ or PIN-ZF_D141N_ proteins. Free FAM label (6-Carboxyfluorescein, C0662 Sigma-Aldrich) was used as a control of affinity determination assay. Unlabeled and HPLC purified hIL-6_81–98_ RNA oligonucleotide was purchased from Sigma-Aldrich. Protein concentrations were determined by measuring the absorbance at 280 nm using a NanoDrop 2000 spectrophotometer (Thermo Scientific). Proteins absorption coefficients were calculated on the basis of amino acid sequence. Samples were prepared using the twofold serial dilution method; thus, in each sample, the concentration of protein gradually changed. The reaction buffer for detection of sample fluorescence contained 25 mM Tris-HCl pH 7.9, 150 mM NaCl, 5% (w/v) glycerol, 2.5 mM MgCl_2_, 1 mM DTT, 0.5 mM EDTA and 0.05 mM ZnCl_2_. Fluorescence signals were collected using the FluoroLog FL3–12 spectrofluorometer (Horiba Jobin Yvon). Excitation and emission wavelengths were 495 nm and 514 nm, respectively. Measurements of fluorescence was performed at 25 °C using a temperature controlled cuvette holder. The dimensions of the quartz cuvette were 3 × 3 mm (Hellma). Dissociation constants (K_d_) were determined using DynaFit software (version 4.07.111, BioKin)^41^. Determination of the binding model was based on residual distribution of fitted curves and standard deviation of determinated dissociation constants. For calculation of the dissociation constants, a sequential binding model was used: ${\rm{N}}+{\rm{P}}+{\rm{P}}\rightleftarrows {\rm{NP}}+{\rm{P}}\rightleftarrows {\rm{NPP}}$ (N – oligonucleotide, P – protein), where K_d1_ and K_d2_ were equal dissociation constants. Additionally, two binding models were tested. The first one was characterized by K_d1_ ≠ K_d2_, and the second one was simplified to the single equation ${\rm{N}}+{\rm{P}}\rightleftarrows {\rm{NP}}$. The graph errors represent standard deviations from 3 independent experiments. For statistical analysis of differences between calculated dissociation constants for oligonucleotide complexes with MCPIP_D141N_, PINZF_D141N_ and PIN_D141N_ one-way ANOVA followed by Tukey’s multiple comparison test was used.

### Gel filtration assays

Analytical size exclusion chromatography was performed using a Superdex 200 Increase 10/300 GL column (GE Healthcare) that was calibrated with the following protein standards: myoglobin, α-chymotrypsinogen, β-lactoglobulin, ovalbumin, bovine serum albumin, apoferritin and thyroglobulin. The apparent molecular weight of MCPIP1 proteins was determined based on the column calibration curve. For determination of homooligomerization of the analyzed samples, multiple Gaussian peak fits were performed for chromatogram data using OriginPro 2017 software (OriginLab).

### Native polyacrylamide gel electrophoresis

Protein samples for native electrophoresis were prepared with the addition of twofold excess of concentrated loading dye that comprised 62.5 mM Tris-HCl, pH 6.8, 25% glycerol, and 1% (w/v) bromophenol blue. The gels contained 300 mM Tris-HCl, pH 8.8, and 6% polyacrylamide (concentrations of acrylamide/bis-acrylamide were 30%/1% w/v). Electrophoresis was performed at 80 V using running buffer containing 25 mM Tris and 192 mM glycine. Gels were stained with Coomassie Brilliant Blue G-250 solution.

### Data availability

The datasets analyzed during the current study are available from the corresponding author on reasonable request.

## Electronic supplementary material

Supplementary file
