# Supplementary material for: Substrate specificity of human MCPIP1 endoribonuclease
Source: Sci Rep. 2018 May 9;8:7381. doi: 10.1038/s41598-018-25765-2 (PMC5943514; doi:10.1038/s41598-018-25765-2)
Supplement: Supplementary file 1 — Supplementary file [file 41598_2018_25765_MOESM1_ESM.pdf]

SUPPLEMENTARY DATA

Substrate specificity of human MCPIP1 endoribonuclease

Mateusz Wilamowski[1], Andrzej Gorecki[2], Marta Dziedzicka-Wasylewska[2], Jolanta Jura[1*]

[1]Department of General Biochemistry, Faculty of Biochemistry, Biophysics and Biotechnology, Jagiellonian University, Krakow, Poland

[2]Department of Physical Biochemistry, Faculty of Biochemistry, Biophysics and Biotechnology, Jagiellonian University, Krakow, Poland

Fig.S1

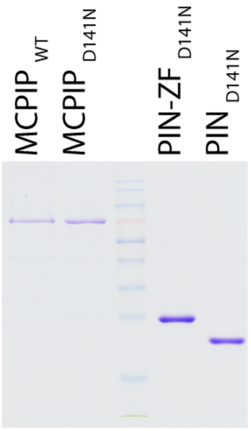

Supplementary Figure S1. SDS-PAGE analysis of purified proteins. One microgram of selected proteins was loaded on a 12% polyacrylamide gel and resolved through electrophoresis. Molecular weights of protein fragments calculated based on the amino acid sequence are as follows: MCPIP1 65.7 kDa; PIN-ZF: 24.7 kDa; PIN: 21.1 kDa. The PageRuler protein ladder (Thermo Scientific) was used, and molecular mass standards from the bottom of the gel are as follows: 10 (green), 15, 25, 35, 40, 55, 70 (red), 100, 130, and 180 (masses in kDa).

Fig.S2A

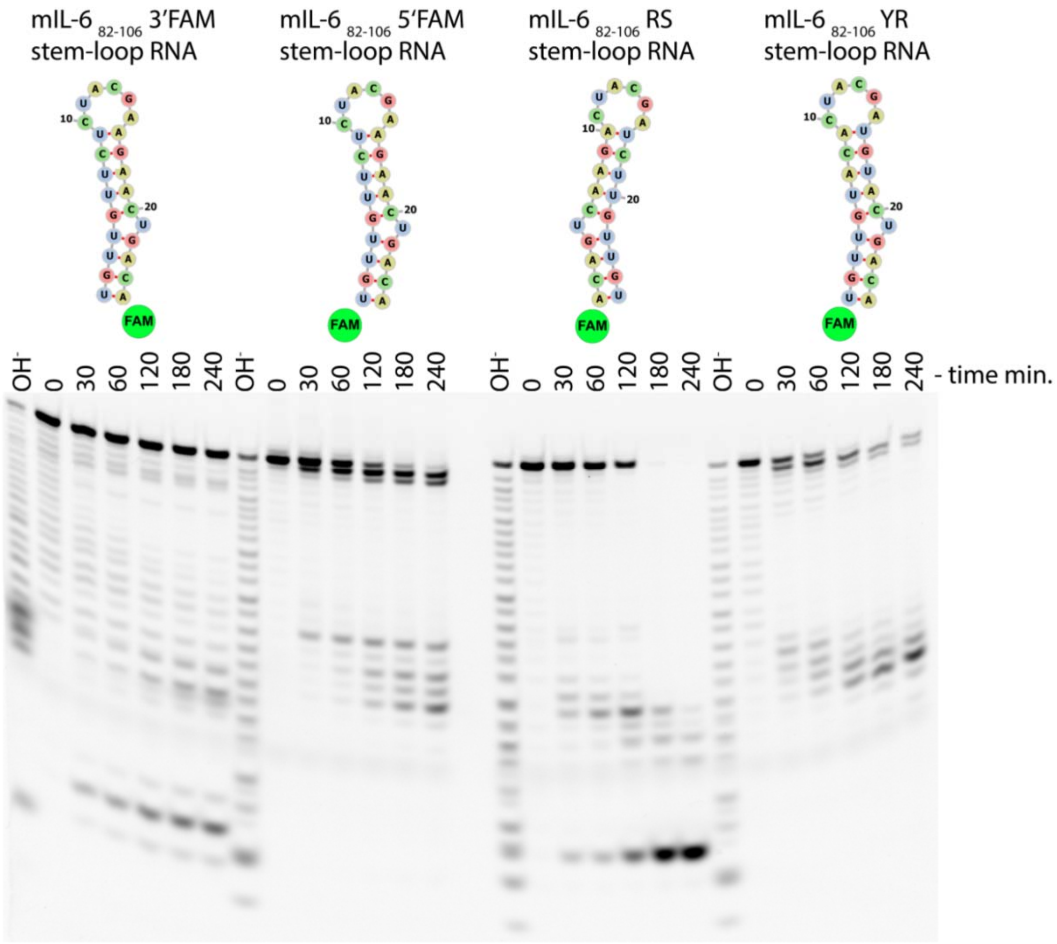

Fig.S2B

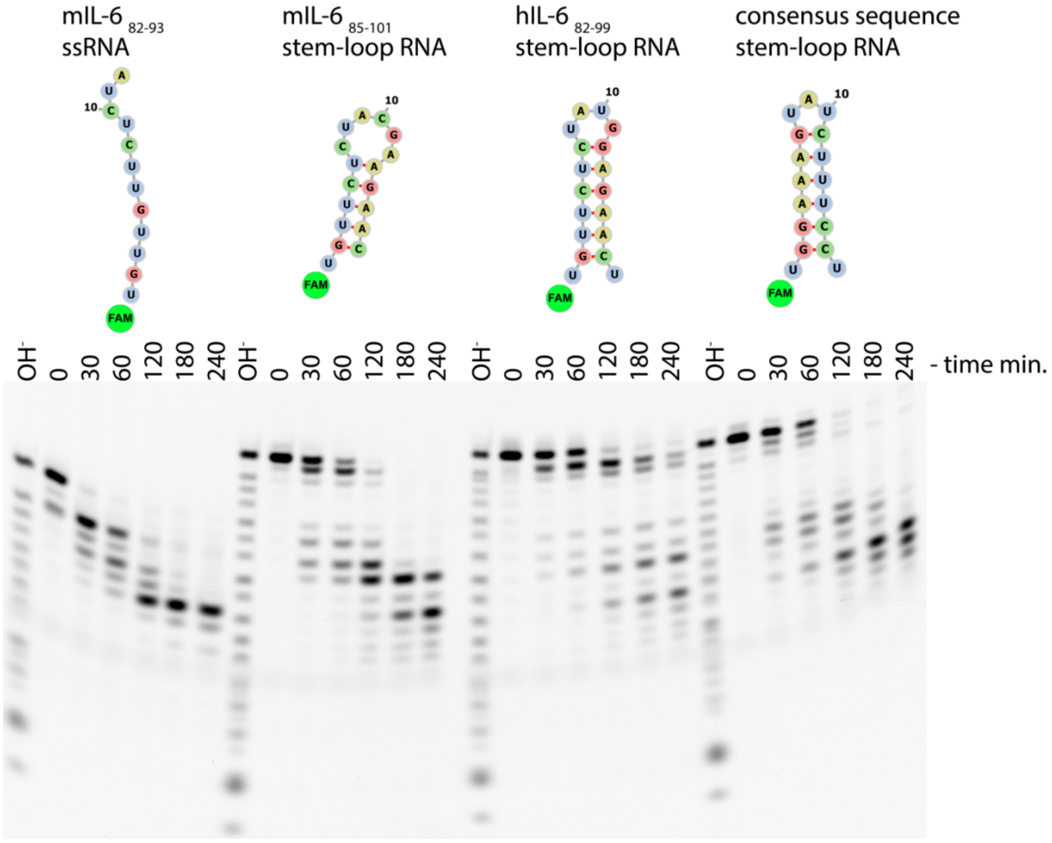

Fig.S2C

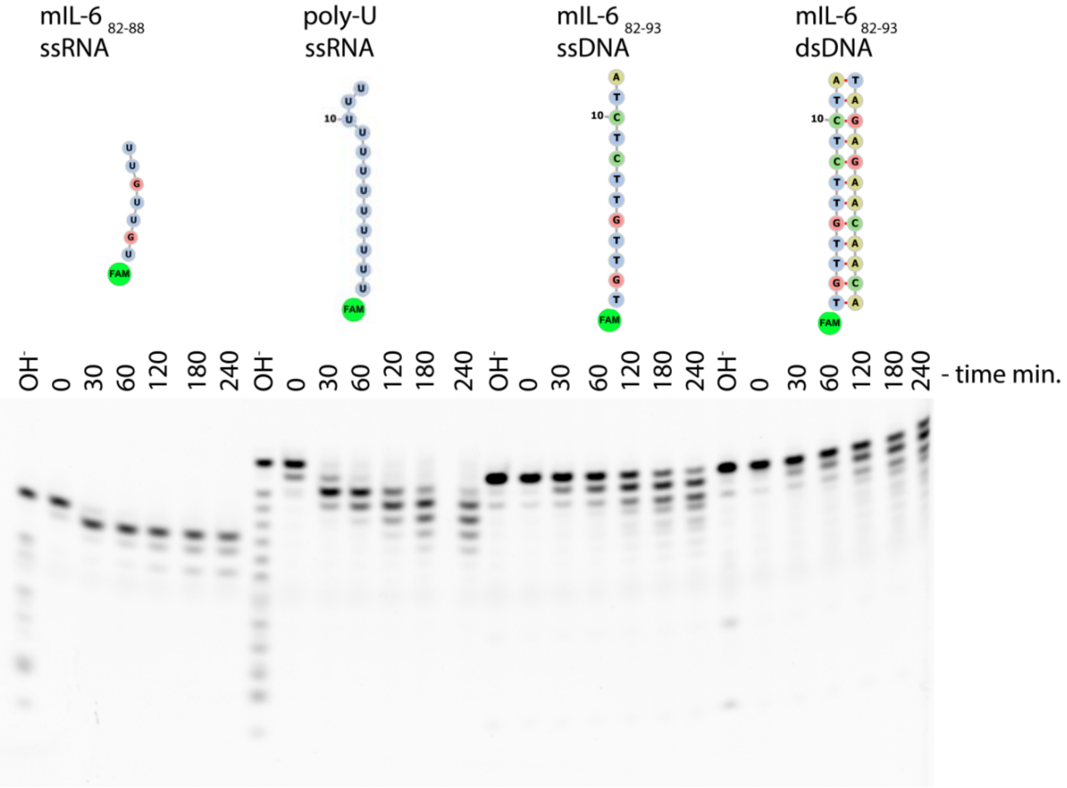

Fig.S2D

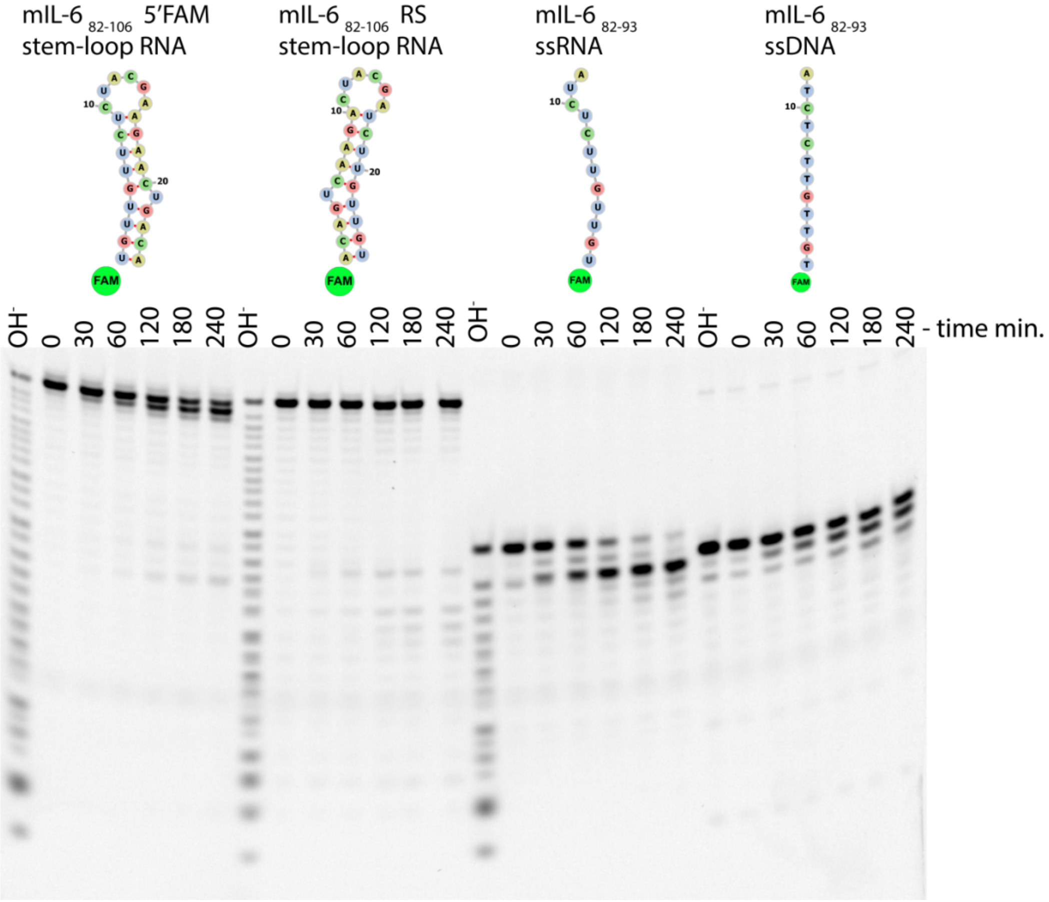

Fig.S2E

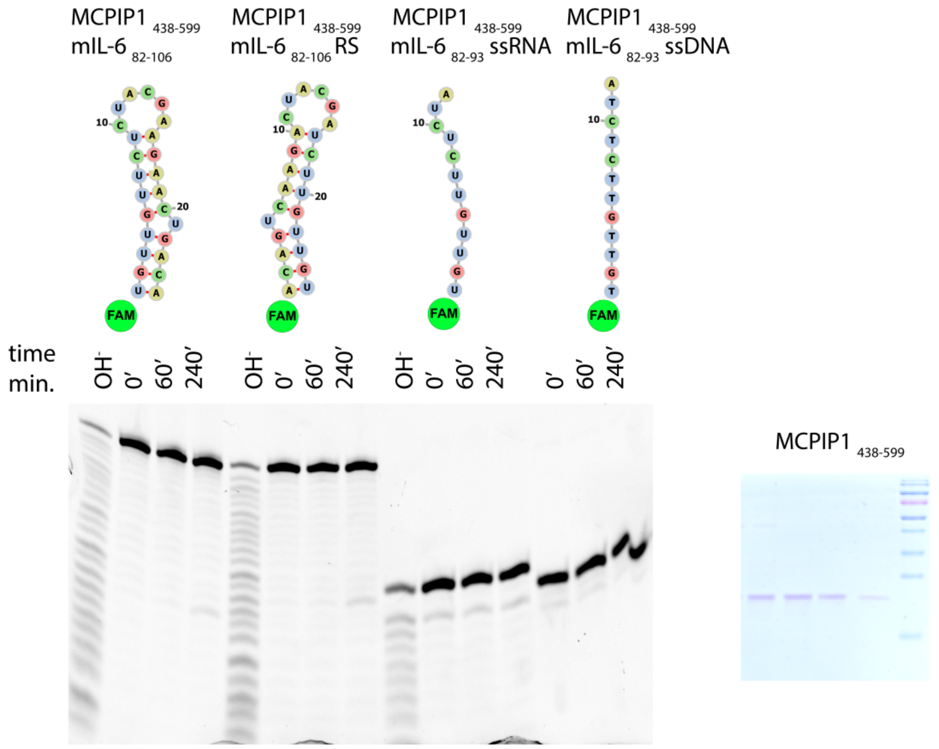

Fig.S2F

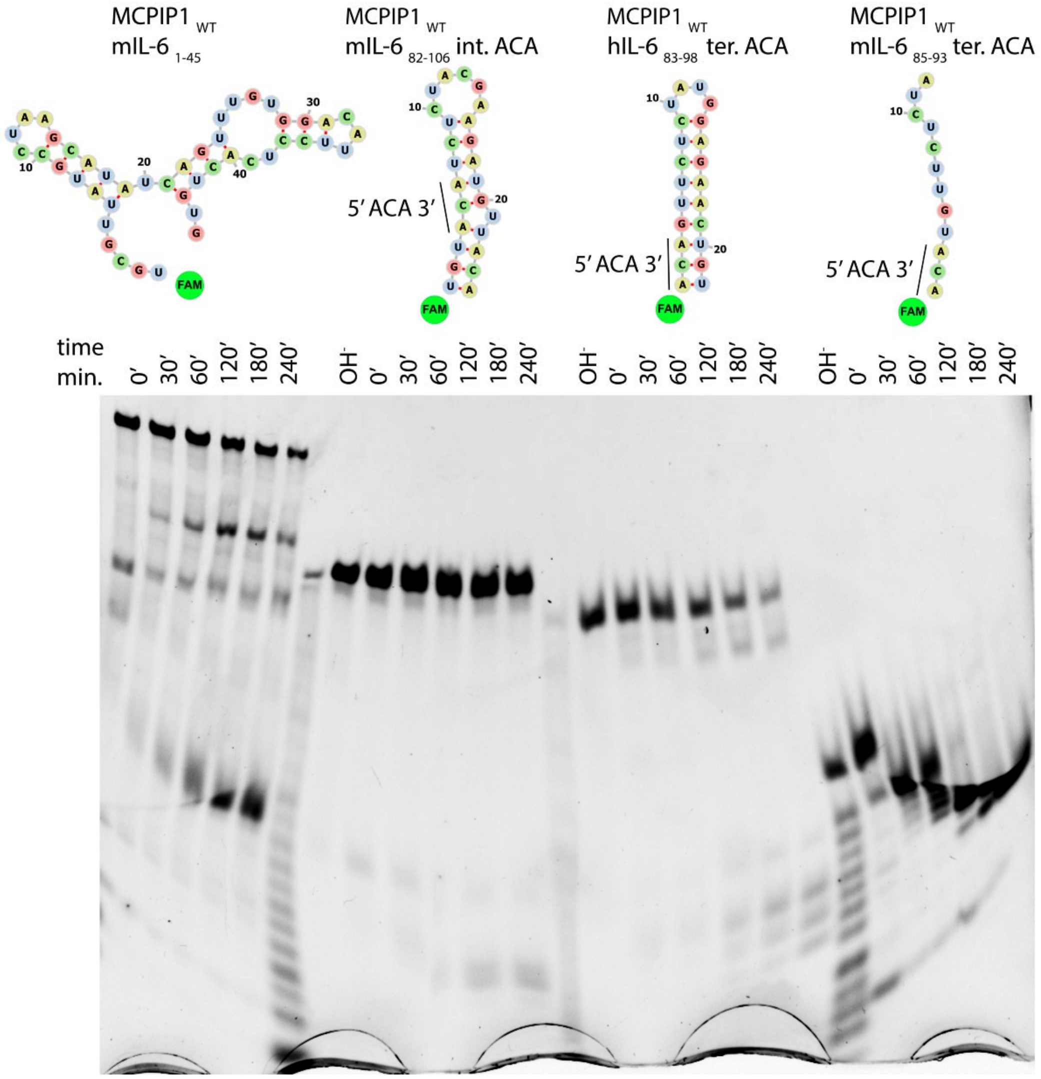

Supplementary Figure S2. RNA fragments obtained upon MCPIP1-catalyzed cleavage. Supplementary Figure S2 illustrated that equal signal acquisition (0.5 sec) and image processing were done to presented in article figures. Figures 1A, 1B, 1C, 1D, 2A, 2B, 3A were made by the separation of gels showed at Supplementary Figure S2. The oligonucleotides were labeled at the 5' or 3' end with FAM dye. Concentrations of the labeled oligonucleotides and MCPIP1 protein were 7.5 µM and 2 µM, respectively. The reaction products were resolved on 20% denaturing PAGE. **A.** MCPIP1 $_{WT}$ catalyzed cleavage of the mIL-6 $_{82\text{-}106}$ 3'FAM, mIL-6 $_{82\text{-}106}$ 5'FAM, mIL-6 $_{82\text{-}106}$ RS, mIL-6 $_{82\text{-}106}$ YR.
B. MCPIP1 $_{WT}$ catalyzed cleavage of the mIL-6 $_{82\text{-}93}$, mIL-6 $_{85\text{-}101}$ short stem, hIL-6 $_{82\text{-}99}$, consensus stem-loop. **C.** MCPIP1 $_{WT}$ catalyzed cleavage of the mIL-6 $_{82\text{-}88}$, poly-U, mIL-6 $_{82\text{-}93}$ ssDNA and mIL-6 $_{82\text{-}93}$ dsDNA. **D.** MCPIP1 $_{D141N}$ catalyzed cleavage of the mIL-6 $_{82\text{-}106}$ 5'FAM, mIL-6 $_{82\text{-}106}$ RS, mIL-6 $_{82\text{-}93}$ ssRNA and mIL-6 $_{82\text{-}93}$ ssDNA oligonucleotides. The D141N mutation of a conserved aspartate of the PIN domain catalytic center of the MCPIP1 decreased its ribonucleolytic activity. **E.** RNA cleavage assay induced by MCPIP1 $_{438\text{-}599}$ protein which do not possess PIN nuclease domain. Right panel shows SDS-PAGE of purified MCPIP1 $_{438\text{-}599}$ protein **F.** MCPIP1 $_{WT}$ catalyzed cleavage of the mIL-6 $_{1\text{-}45}$, mIL-6 $_{82\text{-}106}$ int. ACA, mIL-6 $_{83\text{-}98}$ ter. ACA and mIL-6 $_{85\text{-}93}$ ter. ACA oligonucleotides.

Fig.S3

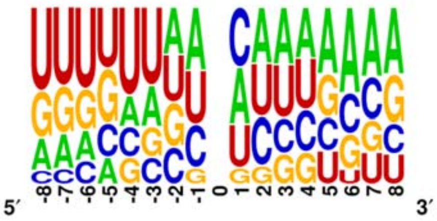

Supplementary Figure S3. Logotypes of oligoribonucleotides sequences cleaved by MCPIP1. Sites of enzymatic hydrolysis of oligonucleotides are marked as 0. Analyzed MCPIP1-induced cleavage sites were collected from RNase assays of all investigated RNA sequences without poly-U sequences.

Supplementary Table S1. Cleavage sites of oligoribonucleotides collected from MCPIP1 RNase assay. Listed cleavage sites were used for preparing the logotype of MCPIP1-triggered cleavage sites (Supplementary Figure S3). Nts that form the loop site of stem-loop structures are underlined.

Oligonucleotide:	Sequence (5'-3'):
**mIL-6 $_{82\text{-}106}$ 3'FAM**	UGUUGUUCUCUACGAAGAACUGAC↓A-FAM
	UGUUGUUCUCUACGAAGAACUGA↓CA-FAM
	UGUUGUUCUCUACGAAGA↓ACUGACA-FAM
	UGUUGUUCUCUACGAAG↓AACUGACA-FAM
**mIL-6 $_{82\text{-}106}$ 5'FAM**	FAM-UGUUGUUCUCUACGAAGAACUGAC↓A
	FAM-UGUUGUUCUCUACGAAGAACUGA↓CA
	FAM-UGUUGUUCUCU↓ACGAAGAACUGACA
	FAM-UGUUGUUCUC↓UACGAAGAACUGACA
	FAM-UGUUGUUCU↓CUACGAAGAACUGACA
	FAM-UGUUGUUC↓UCUACGAAGAACUGACA
	FAM-UGUUGUU↓CUCUACGAAGAACUGACA
	FAM-UGUUGU↓UCUCUACGAAGAACUGACA
**mIL-6 $_{82\text{-}106}$ RS**	FAM-ACAGUCAAGA↓CUACGAUCUUGUUGU
	FAM-ACAGUCAAG↓ACUACGAUCUUGUUGU
	FAM-ACAGUCAA↓GACUACGAUCUUGUUGU
	FAM-ACAGUCA↓AGACUACGAUCUUGUUGU
	FAM-A↓CAGUCAAGACUACGAUCUUGUUGU
**mIL-6 $_{82\text{-}106}$ YR**	FAM-UGUUGUACACUACGAUGUACUGAC↓A
	FAM-UGUUGUACACUACGAUGUACUGA↓CA
	FAM-UGUUGUACA↓CUACGAUGUACUGACA
	FAM-UGUUGUAC↓ACUACGAUGUACUGACA
	FAM-UGUUGUA↓CACUACGAUGUACUGACA
	FAM-UGUUGU↓ACACUACGAUGUACUGACA
**mIL-6 $_{85\text{-}101}$ short stem**	FAM-UGUUCUCUACGAAGAA↓C
	FAM-UGUUCUCUACGAAGA↓AC
	FAM-UGUUCUCUACGA↓AGAAC
	FAM-UGUUCUCUACG↓AAGAAC
	FAM-UGUUCUCUAC↓GAAGAAC
	FAM-UGUUCUCUA↓CGAAGAAC
	FAM-UGUUCUCU↓ACGAAGAAC
	FAM-UGUUCUC↓UACGAAGAAC
	FAM-UGUUCU↓CUACGAAGAAC
**hIL-6 $_{82\text{-}99}$**	FAM-UGUUCUCUAUGGAGAA↓CU
	FAM-UGUUCUCUAUGGAGA↓ACU
	FAM-UGUUCUCUAUG↓GAGAACU
	FAM-UGUUCUCUAU↓GGAGAACU

	FAM−UGUUCUCUA↓UGGAGAACU
	FAM−UGUUCUCU↓AUGGAGAACU
	FAM−UGUUCUC↓UAUGGAGAACU
	FAM−UGUUCU↓CUAUGGAGAACU
**consensus stem-loop**	FAM−UGGAAAGUAUCUUUCC↓U
	FAM−UGGAAAGUAUCUUUC↓CU
	FAM−UGGAAAGUAU↓CUUUCCU
	FAM−UGGAAAGUA↓UCUUUCCU
	FAM−UGGAAAGU↓AUCUUUCCU
	FAM−UGGAAAG↓UAUCUUUCCU
	FAM−UGGAAA↓GUAUCUUUCCU
**mIL-6 $_{82\text{-}93}$**	FAM−UGUUGUUCUC↓UA
	FAM−UGUUGUUCU↓CUA
	FAM−UGUUGUUC↓UCUA
	FAM−UGUUGUU↓CUCUA
	FAM−UGUUGU↓UCUCUA
**mIL-6 $_{82\text{-}88}$**	FAM−UGUUGU↓U
**poly-U**	FAM−UUUUUUUUUUU↓U
	FAM−UUUUUUUUUU↓UU
	FAM−UUUUUUUUU↓UUU
	FAM−UUUUUUUU↓UUUU

Fig.S4A

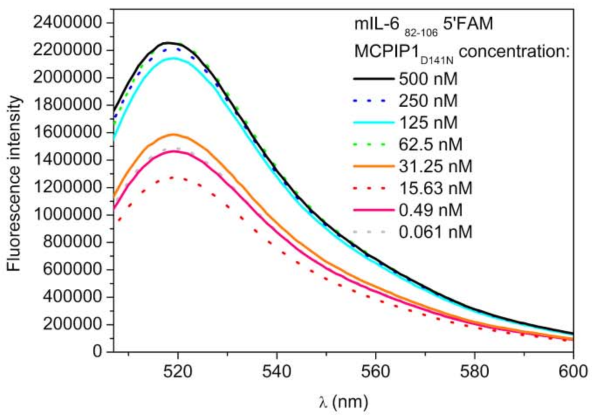

Fig.S4B

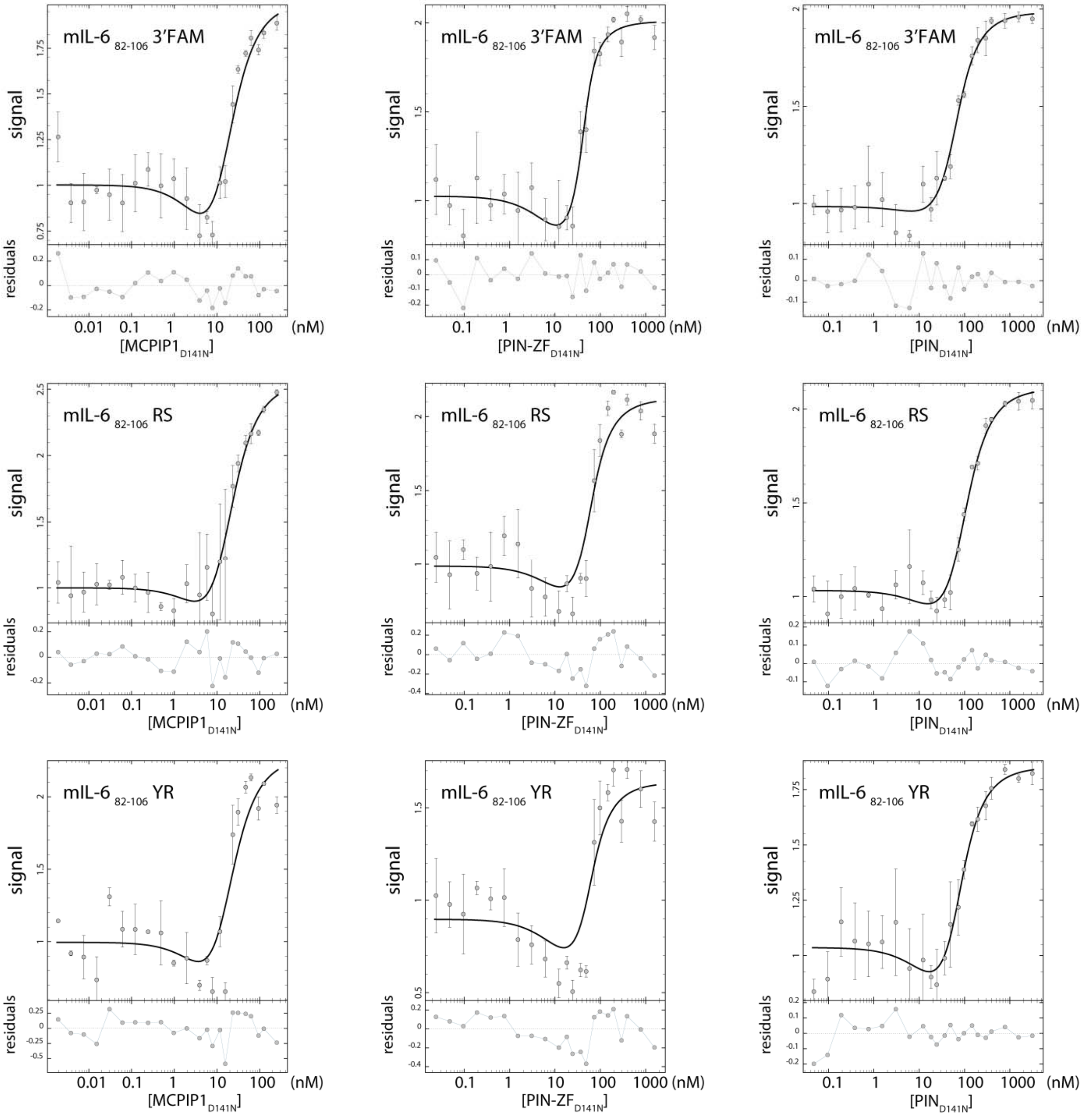

Fig.S4C

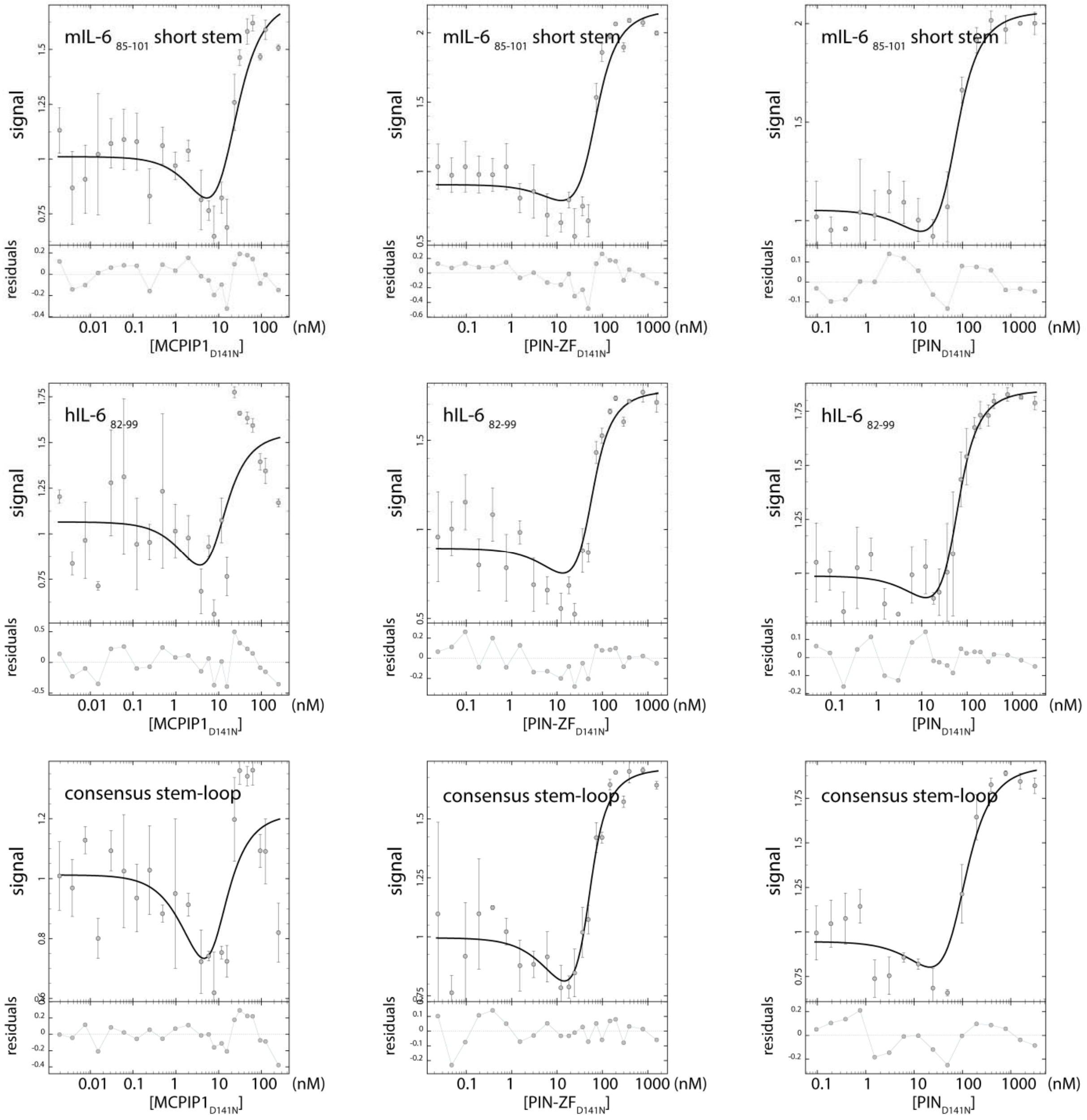
mIL-6 85-101 short stem
hIL-6 82-99
consensus stem-loop
signal
residuals
(nM)
[MCPIP1 D141N]
[PIN-ZF D141N]
[PIN D141N]

Fig.S4D

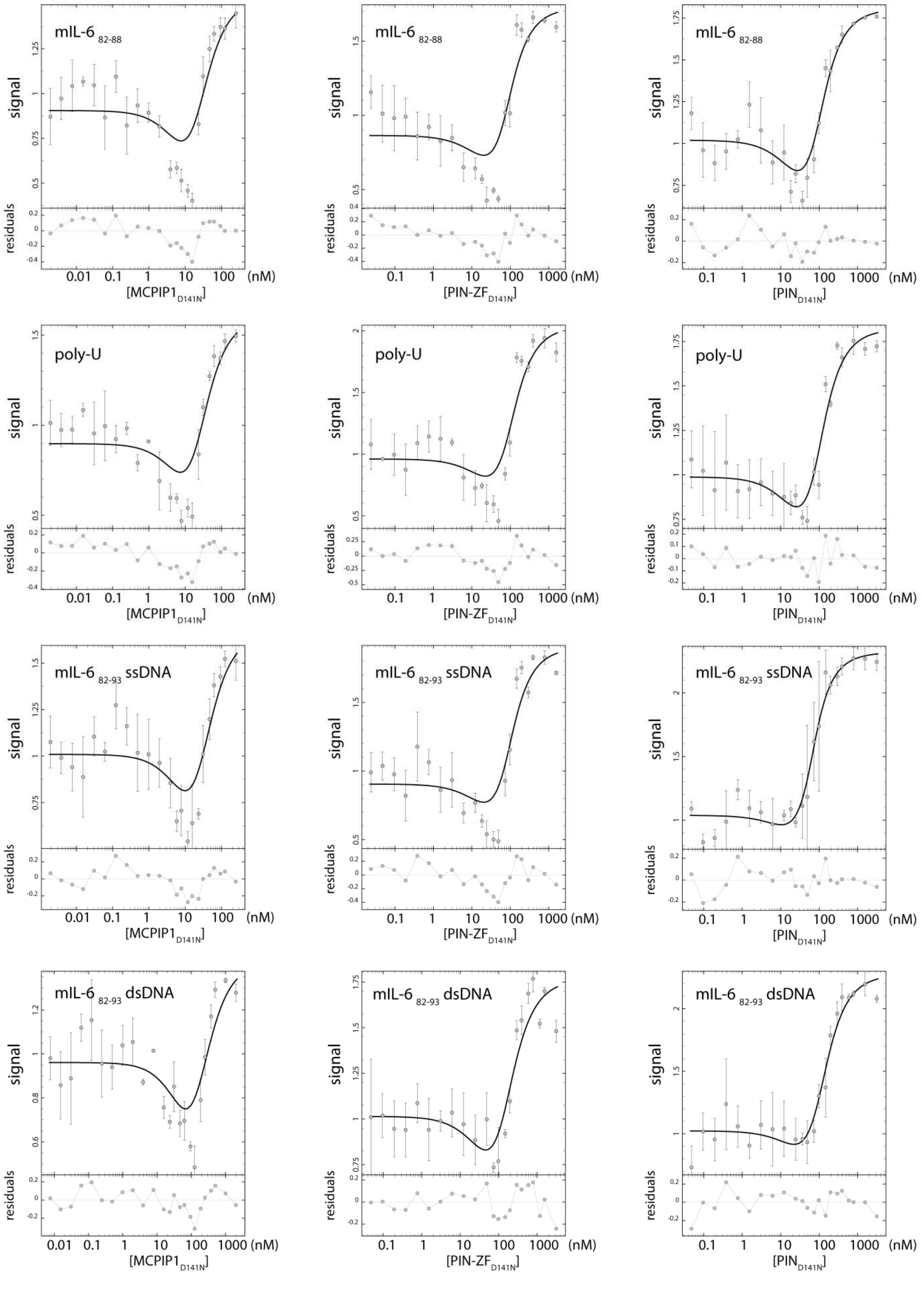
mIL-6 82-88
mIL-6 82-88
mIL-6 82-88
poly-U
poly-U
poly-U
mIL-6 82-93 ssDNA
mIL-6 82-93 ssDNA
mIL-6 82-93 ssDNA
mIL-6 82-93 dsDNA
mIL-6 82-93 dsDNA
mIL-6 82-93 dsDNA
signal
residuals
(nM)
[MCPIP1 D141N]
[PIN-ZF D141N]
[PIN D141N]

Supplementary Figure S4. Graphs present the data obtained in affinity determination assays. **A.** Representative fluorescence emission spectra of the mIL-6 $_{82\text{-}106}$ 5'FAM and mIL-6 $_{82\text{-}93}$ in a presence of various MCPIP1 $_{D141N}$ concentrations. Concentration of oligonucleotides were 2 nM, which are the same as for affinity determination assay. Samples were measured in buffer 25 mM Tris-HCl pH 7.9, 150 mM NaCl, 5% (w/v) glycerol, 2.5 mM $MgCl_2$, 1 mM DTT, 0.5 mM EDTA and 0.05 mM $ZnCl_2$. Samples were excited at 495 nm. **B.** Graphs illustrate the interaction of proteins (MCPIP1 $_{D141N}$, PIN $_{D141N}$, and PIN-ZF $_{D141N}$) with oligonucleotides forming 25-nt-long stem-loop RNA structures. **C.** Interaction with oligonucleotides forming 17-18-nt-long stem-loop RNA structures. **D.** Interaction with oligonucleotides forming 7-12-nt-long ssRNA, ssDNA and dsDNA. Functions were fitted to the fluorescence intensity data points using the sequential binding model N + P + P ⇄ NP + P ⇄ NPP (N – oligonucleotide, P – protein). The depicted errors bars are standard deviations, n = 3.

Fig.S5A

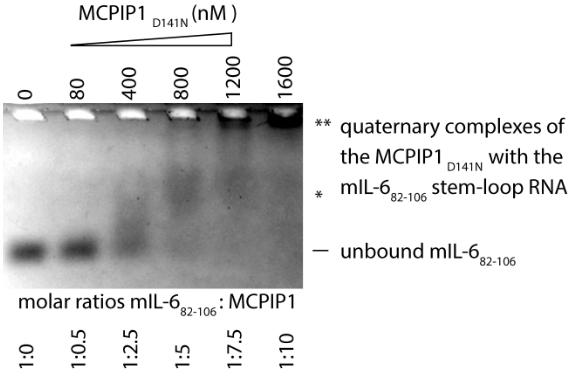

Fig.S5B

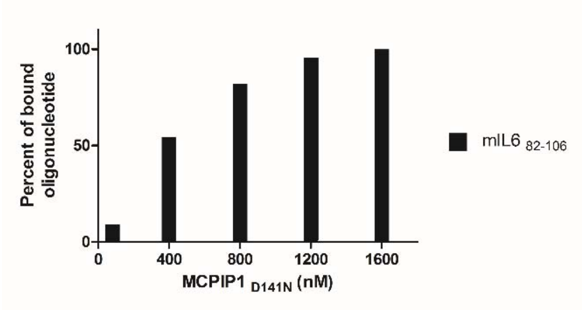

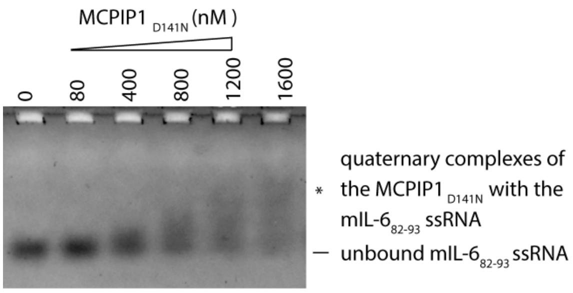

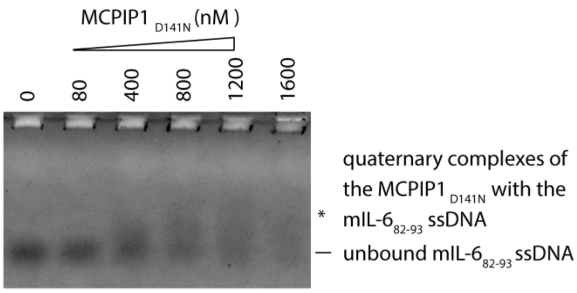

Supplementary Figure S5. EMSA of the MCPIP1 $_{D141N}$ complexes with stem-loop RNA, ssRNA and ssDNA oligonucleotides. **A.** Samples of MCPIP1 $_{D141N}$ complexes with different oligonucleotides: mIL-

6 $_{82\text{-}106}$ 5'FAM, mIL-6 $_{82\text{-}93}$ ssRNA and mIL-6 $_{82\text{-}93}$ ssDNA were incubated 30 minutes in buffer containing 25 mM Tris-HCl pH 7.9, 150 mM NaCl, 5% (w/v) glycerol, 2.5 mM $MgCl_2$, 1 mM DTT, 0.5 mM EDTA and 0.05 mM $ZnCl_2$. Electrophoresis were carried out in TBE buffer using 1% agarose gel.

B. Percentages of bound oligonucleotides fraction were quantified using densitometry analysis of the EMSA results.

Supplementary Table S2. Calculated values of apparent dissociation constants of the complexes of MCPIP1 $_{D141N}$ with oligonucleotides. Apparent Kd were based on EMSA assay showed at Supplementary Figure S5. Kd calculations were based on densitometry analysis obtained from EMSA assay followed by Hill plot analysis of remaining unbound oligonucleotides at different MCPIP1 $_{D141N}$ concentrations.

Protein	Oligonucleotide	Apparent Kd based on EMSA
MCPIP1 $_{D141N}$	mIL-6 $_{82\text{-}106}$ 5'FAM	394 ± 45 (nM)
MCPIP1 $_{D141N}$	3'UTR of the C/EBPβ $_{-60\text{-}624}$ (Lipert et al. 2017)	1580 ± 330 (nM)
MCPIP1 $_{D141N}$	3'UTR of the C/EBPβ $_{15\text{-}270}$ (Lipert et al. 2017)	640 ± 480 (nM)
MCPIP1 $_{D141N}$	3'UTR of the C/EBPβ $_{268\text{-}450}$ (Lipert et al. 2017)	690 ± 330 (nM)
